# Supplementary material for: A Robust One-Step Recombineering System for Enterohemorrhagic Escherichia coli
Source: Microorganisms. 2022 Aug 23;10(9):1689. doi: 10.3390/microorganisms10091689 (PMC9504302; doi:10.3390/microorganisms10091689)
Supplement: Supplementary file 1 [file microorganisms-10-01689-s001.zip › microorganisms-1841403-supplementary.pdf]

microorganisms

Article

A Robust One-Step Recombineering System for Enterohemorrhagic *Escherichia coli*

Lang Peng [1,†], Rexford Mawunyo Dumevi [1,†], Marco Chitto [1], Nadja Haarmann [2], Petya Berger [1,3], Gerald Koudelka [4], Herbert Schmidt [2], Alexander Mellmann [1,3], Ulrich Dobrindt [1] and Michael Berger [1,*]

[1] Institute of Hygiene, University of Münster, 48149 Münster, Germany
[2] Institute of Food Science and Biotechnology, University of Hohenheim, 70599 Stuttgart, Germany
[3] National Consulting Laboratory for Hemolytic Uremic Syndrome (HUS), 48149 Münster, Germany
[4] Department of Biological Sciences, University at Buffalo, Buffalo, NY 14203, USA
* Correspondence: michael.berger@ukmuenster.de; Tel.: +49-251-83-35403
† These authors contributed equally to this work.

Supplementary information

Supplementary Figures

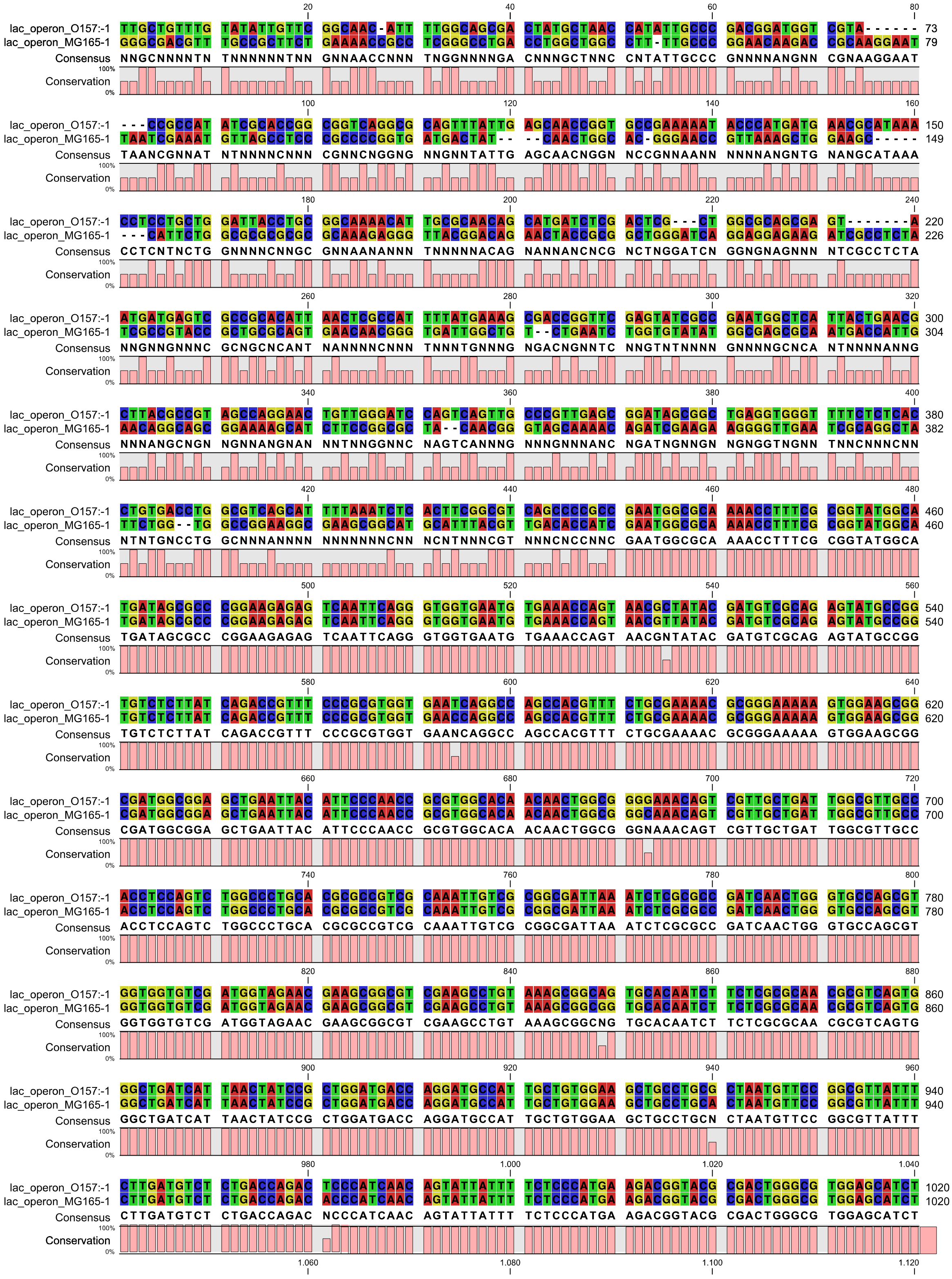

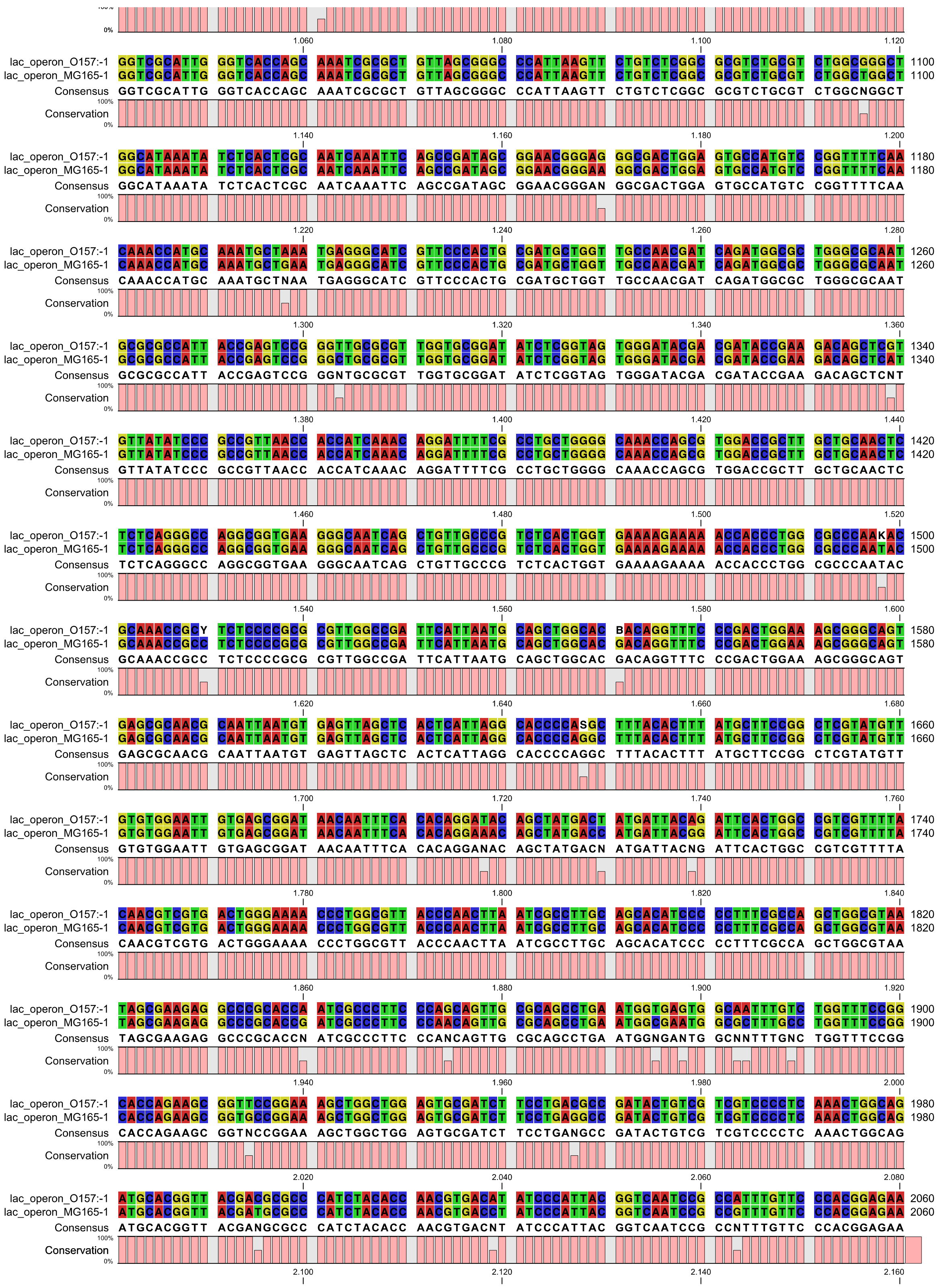

lac_operon_O157:-1 GGTCGCATTG GGTCACCAGC AAATCGCGCT GTTAGCGGGC CCATTAAGTT CTGTCTCGGC GCGTCTGCGT CTGGCGGGCT 1100
lac_operon_MG165-1 GGTCGCATTG GGTCACCAGC AAATCGCGCT GTTAGCGGGC CCATTAAGTT CTGTCTCGGC GCGTCTGCGT CTGGCTGGCT 1100
Consensus GGTCGCATTG GGTCACCAGC AAATCGCGCT GTTAGCGGGC CCATTAAGTT CTGTCTCGGC GCGTCTGCGT CTGGCNGGCT
Conservation
lac_operon_O157:-1 GGCATAAATA TCTCACTCGC AATCAAATTC AGCCGATAGC GGAACGGGAG GGCGACTGGA GTGCCATGTC CGGTTTTCAA 1180
lac_operon_MG165-1 GGCATAAATA TCTCACTCGC AATCAAATTC AGCCGATAGC GGAACGGGAA GGCGACTGGA GTGCCATGTC CGGTTTTCAA 1180
Consensus GGCATAAATA TCTCACTCGC AATCAAATTC AGCCGATAGC GGAACGGGAN GGCGACTGGA GTGCCATGTC CGGTTTTCAA
Conservation
lac_operon_O157:-1 CAAACCATGC AAATGCTAAA TGAGGGCATC GTTCCCACTG CGATGCTGGT TGCCAACGAT CAGATGGCGC TGGGCGCAAT 1260
lac_operon_MG165-1 CAAACCATGC AAATGCTGAA TGAGGGCATC GTTCCCACTG CGATGCTGGT TGCCAACGAT CAGATGGCGC TGGGCGCAAT 1260
Consensus CAAACCATGC AAATGCTNAA TGAGGGCATC GTTCCCACTG CGATGCTGGT TGCCAACGAT CAGATGGCGC TGGGCGCAAT
Conservation
lac_operon_O157:-1 GCGCGCCATT ACCGAGTCCG GGTTGCGCGT TGGTGCGGAT ATCTCGGTAG TGGGATACGA CGATACCGAA GACAGCTCGT 1340
lac_operon_MG165-1 GCGCGCCATT ACCGAGTCCG GGCTGCGCGT TGGTGCGGAT ATCTCGGTAG TGGGATACGA CGATACCGAA GACAGCTCAT 1340
Consensus GCGCGCCATT ACCGAGTCCG GGNTGCGCGT TGGTGCGGAT ATCTCGGTAG TGGGATACGA CGATACCGAA GACAGCTCNT
Conservation
lac_operon_O157:-1 GTTATATCCC GCCGTTAACC ACCATCAAAC AGGATTTTCG CCTGCTGGGG CAAACCAGCG TGGACCGCTT GCTGCAACTC 1420
lac_operon_MG165-1 GTTATATCCC GCCGTTAACC ACCATCAAAC AGGATTTTCG CCTGCTGGGG CAAACCAGCG TGGACCGCTT GCTGCAACTC 1420
Consensus GTTATATCCC GCCGTTAACC ACCATCAAAC AGGATTTTCG CCTGCTGGGG CAAACCAGCG TGGACCGCTT GCTGCAACTC
Conservation
lac_operon_O157:-1 TCTCAGGGCC AGGCGGTGAA GGGCAATCAG CTGTTGCCCG TCTCACTGGT GAAAAGAAAA ACCACCCTGG CGCCCAAKAC 1500
lac_operon_MG165-1 TCTCAGGGCC AGGCGGTGAA GGGCAATCAG CTGTTGCCCG TCTCACTGGT GAAAAGAAAA ACCACCCTGG CGCCCAATAC 1500
Consensus TCTCAGGGCC AGGCGGTGAA GGGCAATCAG CTGTTGCCCG TCTCACTGGT GAAAAGAAAA ACCACCCTGG CGCCCAATAC
Conservation
lac_operon_O157:-1 GCAAACCGCY TCTCCCCGCG CGTTGGCCGA TTCATTAATG CAGCTGGCAC BACAGGTTTC CCGACTGGAA AGCGGGCAGT 1580
lac_operon_MG165-1 GCAAACCGCC TCTCCCCGCG CGTTGGCCGA TTCATTAATG CAGCTGGCAC GACAGGTTTC CCGACTGGAA AGCGGGCAGT 1580
Consensus GCAAACCGCC TCTCCCCGCG CGTTGGCCGA TTCATTAATG CAGCTGGCAC GACAGGTTTC CCGACTGGAA AGCGGGCAGT
Conservation
lac_operon_O157:-1 GAGCGCAACG CAATTAATGT GAGTTAGCTC ACTCATTAGG CACCCCASGC TTTACACTTT ATGCTTCCGG CTCGTATGTT 1660
lac_operon_MG165-1 GAGCGCAACG CAATTAATGT GAGTTAGCTC ACTCATTAGG CACCCCAGGC TTTACACTTT ATGCTTCCGG CTCGTATGTT 1660
Consensus GAGCGCAACG CAATTAATGT GAGTTAGCTC ACTCATTAGG CACCCCAGGC TTTACACTTT ATGCTTCCGG CTCGTATGTT
Conservation
lac_operon_O157:-1 GTGTGGAATT GTGAGCGGAT AACAATTTCA CACAGGATAC AGCTATGACT ATGATTACAG ATTCACTGGC CGTCGTTTTA 1740
lac_operon_MG165-1 GTGTGGAATT GTGAGCGGAT AACAATTTCA CACAGGAAAC AGCTATGACC ATGATTACGG ATTCACTGGC CGTCGTTTTA 1740
Consensus GTGTGGAATT GTGAGCGGAT AACAATTTCA CACAGGANAC AGCTATGACN ATGATTACNG ATTCACTGGC CGTCGTTTTA
Conservation
lac_operon_O157:-1 CAACGTCGTG ACTGGGAAAA CCCTGGCGTT ACCCAACTTA ATCGCCTTGC AGCACATCCC CCTTTCGCCA GCTGGCGTAA 1820
lac_operon_MG165-1 CAACGTCGTG ACTGGGAAAA CCCTGGCGTT ACCCAACTTA ATCGCCTTGC AGCACATCCC CCTTTCGCCA GCTGGCGTAA 1820
Consensus CAACGTCGTG ACTGGGAAAA CCCTGGCGTT ACCCAACTTA ATCGCCTTGC AGCACATCCC CCTTTCGCCA GCTGGCGTAA
Conservation
lac_operon_O157:-1 TAGCGAAGAG GCCCGCACCA ATCGCCCTTC CCAGCAGTTG CGCAGCCTGA ATGGTGAGTG GCAATTTGTC TGGTTTCCGG 1900
lac_operon_MG165-1 TAGCGAAGAG GCCCGCACCG ATCGCCCTTC CCAACAGTTG CGCAGCCTGA ATGGCGAATG GCGCTTTGCC TGGTTTCCGG 1900
Consensus TAGCGAAGAG GCCCGCACCN ATCGCCCTTC CCANCAGTTG CGCAGCCTGA ATGGNGANTG GCNNTTTGNC TGGTTTCCGG
Conservation
lac_operon_O157:-1 CACCAGAAGC GGTTCCGGAA AGCTGGCTGG AGTGCGATCT TCCTGACGCC GATACTGTCG TCGTCCCCTC AAACTGGCAG 1980
lac_operon_MG165-1 CACCAGAAGC GGTGCCGGAA AGCTGGCTGG AGTGCGATCT TCCTGAGGCC GATACTGTCG TCGTCCCCTC AAACTGGCAG 1980
Consensus CACCAGAAGC GGTNCCGGAA AGCTGGCTGG AGTGCGATCT TCCTGANGCC GATACTGTCG TCGTCCCCTC AAACTGGCAG
Conservation
lac_operon_O157:-1 ATGCACGGTT ACGACGCGCC CATCTACACC AACGTGACAT ATCCCATTAC GGTCAATCCG CCATTTGTTC CCACGGAGAA 2060
lac_operon_MG165-1 ATGCACGGTT ACGATGCGCC CATCTACACC AACGTGACCT ATCCCATTAC GGTCAATCCG CCGTTTGTTC CCACGGAGAA 2060
Consensus ATGCACGGTT ACGANGCGCC CATCTACACC AACGTGACNT ATCCCATTAC GGTCAATCCG CCNTTTGTTC CCACGGAGAA
Conservation

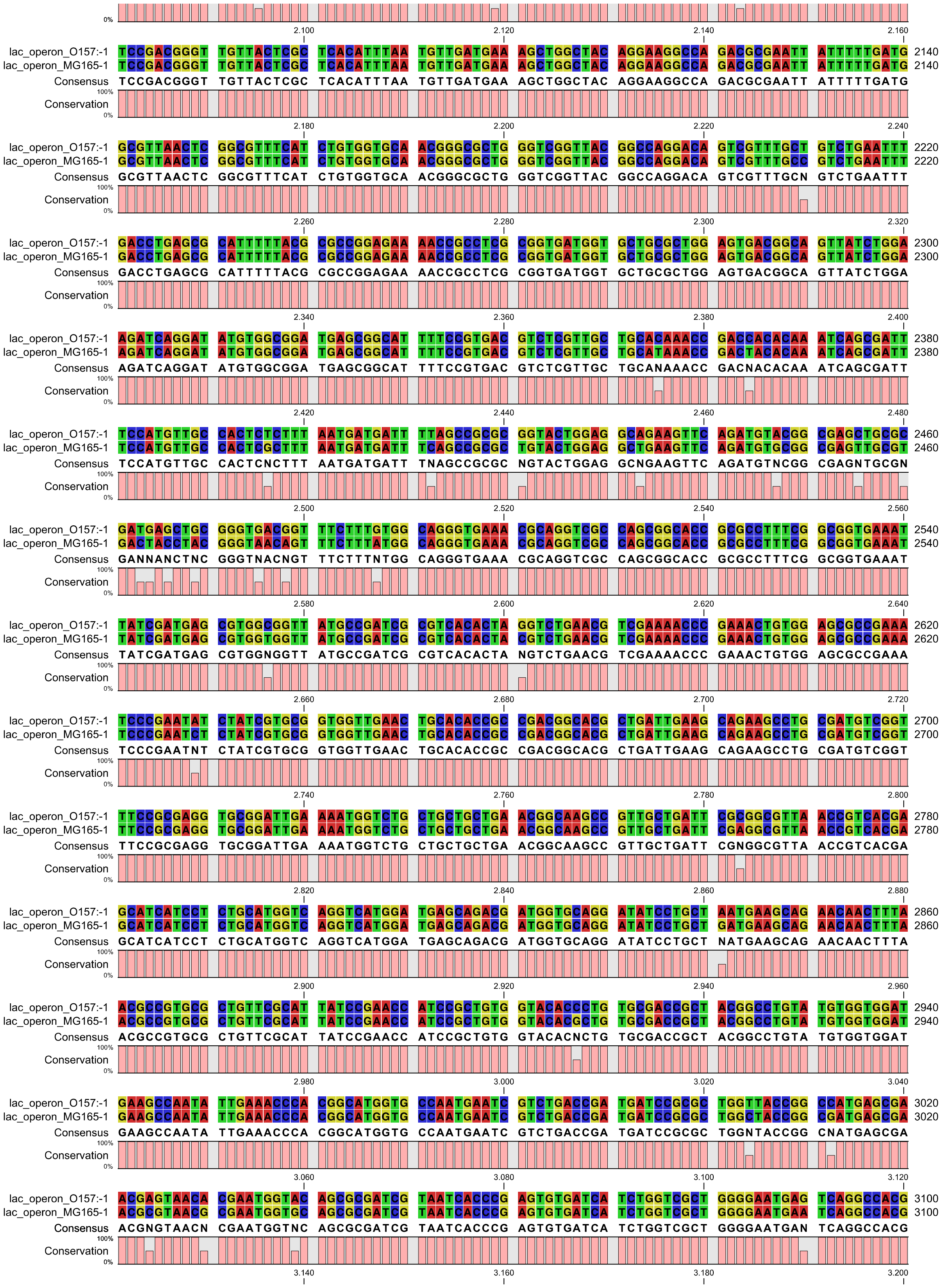
lac_operon_O157:-1 TCCGACGGGT TGTTACTCGC TCACATTTAA TGTTGATGAA AGCTGGCTAC AGGAAGGCCA GACGCGAATT ATTTTTGATG 2140
lac_operon_MG165-1 TCCGACGGGT TGTTACTCGC TCACATTTAA TGTTGATGAA AGCTGGCTAC AGGAAGGCCA GACGCGAATT ATTTTTGATG 2140
Consensus TCCGACGGGT TGTTACTCGC TCACATTTAA TGTTGATGAA AGCTGGCTAC AGGAAGGCCA GACGCGAATT ATTTTTGATG
Conservation
lac_operon_O157:-1 GCGTTAACTC GGCGTTTCAT CTGTGGTGCA ACGGGCGCTG GGTCGGTTAC GGCCAGGACA GTCGTTTGCT GTCTGAATTT 2220
lac_operon_MG165-1 GCGTTAACTC GGCGTTTCAT CTGTGGTGCA ACGGGCGCTG GGTCGGTTAC GGCCAGGACA GTCGTTTGCC GTCTGAATTT 2220
Consensus GCGTTAACTC GGCGTTTCAT CTGTGGTGCA ACGGGCGCTG GGTCGGTTAC GGCCAGGACA GTCGTTTGCN GTCTGAATTT
Conservation
lac_operon_O157:-1 GACCTGAGCG CATTTTTACG CGCCGGAGAA AACCGCCTCG CGGTGATGGT GCTGCGCTGG AGTGACGGCA GTTATCTGGA 2300
lac_operon_MG165-1 GACCTGAGCG CATTTTTACG CGCCGGAGAA AACCGCCTCG CGGTGATGGT GCTGCGCTGG AGTGACGGCA GTTATCTGGA 2300
Consensus GACCTGAGCG CATTTTTACG CGCCGGAGAA AACCGCCTCG CGGTGATGGT GCTGCGCTGG AGTGACGGCA GTTATCTGGA
Conservation
lac_operon_O157:-1 AGATCAGGAT ATGTGGCGGA TGAGCGGCAT TTTCCGTGAC GTCTCGTTGC TGCACAAACC GACCACACAA ATCAGCGATT 2380
lac_operon_MG165-1 AGATCAGGAT ATGTGGCGGA TGAGCGGCAT TTTCCGTGAC GTCTCGTTGC TGCATAAACC GACTACACAA ATCAGCGATT 2380
Consensus AGATCAGGAT ATGTGGCGGA TGAGCGGCAT TTTCCGTGAC GTCTCGTTGC TGCANAAACC GACNACACAA ATCAGCGATT
Conservation
lac_operon_O157:-1 TCCATGTTGC CACTCTCTTT AATGATGATT TTAGCCGCGC GGTACTGGAG GCAGAAGTTC AGATGTACGG CGAGCTGCGC 2460
lac_operon_MG165-1 TCCATGTTGC CACTCGCTTT AATGATGATT TCAGCCGCGC TGTACTGGAG GCTGAAGTTC AGATGTGCGG CGAGTTGCGT 2460
Consensus TCCATGTTGC CACTCNCTTT AATGATGATT TNAGCCGCGC NGTACTGGAG GCNGAAGTTC AGATGTNCGG CGAGNTGCGN
Conservation
lac_operon_O157:-1 GATGAGCTGC GGGTGACGGT TTCTTTGTGG CAGGGTGAAA CGCAGGTCGC CAGCGGCACC GCGCCTTTCG GCGGTGAAAT 2540
lac_operon_MG165-1 GACTACCTAC GGGTAACAGT TTCTTTATGG CAGGGTGAAA CGCAGGTCGC CAGCGGCACC GCGCCTTTCG GCGGTGAAAT 2540
Consensus GANNANCTNC GGGTNACNGT TTCTTTNTGG CAGGGTGAAA CGCAGGTCGC CAGCGGCACC GCGCCTTTCG GCGGTGAAAT
Conservation
lac_operon_O157:-1 TATCGATGAG CGTGGCGGTT ATGCCGATCG CGTCACACTA GGTCTGAACG TCGAAAACCC GAAACTGTGG AGCGCCGAAA 2620
lac_operon_MG165-1 TATCGATGAG CGTGGTGGTT ATGCCGATCG CGTCACACTA CGTCTGAACG TCGAAAACCC GAAACTGTGG AGCGCCGAAA 2620
Consensus TATCGATGAG CGTGGNGGTT ATGCCGATCG CGTCACACTA NGTCTGAACG TCGAAAACCC GAAACTGTGG AGCGCCGAAA
Conservation
lac_operon_O157:-1 TCCCGAATAT CTATCGTGCG GTGGTTGAAC TGCACACCGC CGACGGCACG CTGATTGAAG CAGAAGCCTG CGATGTCGGT 2700
lac_operon_MG165-1 TCCCGAATCT CTATCGTGCG GTGGTTGAAC TGCACACCGC CGACGGCACG CTGATTGAAG CAGAAGCCTG CGATGTCGGT 2700
Consensus TCCCGAATNT CTATCGTGCG GTGGTTGAAC TGCACACCGC CGACGGCACG CTGATTGAAG CAGAAGCCTG CGATGTCGGT
Conservation
lac_operon_O157:-1 TTCCGCGAGG TGCGGATTGA AAATGGTCTG CTGCTGCTGA ACGGCAAGCC GTTGCTGATT CGCGGCGTTA ACCGTCACGA 2780
lac_operon_MG165-1 TTCCGCGAGG TGCGGATTGA AAATGGTCTG CTGCTGCTGA ACGGCAAGCC GTTGCTGATT CGAGGCGTTA ACCGTCACGA 2780
Consensus TTCCGCGAGG TGCGGATTGA AAATGGTCTG CTGCTGCTGA ACGGCAAGCC GTTGCTGATT CGNGGCGTTA ACCGTCACGA
Conservation
lac_operon_O157:-1 GCATCATCCT CTGCATGGTC AGGTCATGGA TGAGCAGACG ATGGTGCAGG ATATCCTGCT AATGAAGCAG AACAACTTTA 2860
lac_operon_MG165-1 GCATCATCCT CTGCATGGTC AGGTCATGGA TGAGCAGACG ATGGTGCAGG ATATCCTGCT GATGAAGCAG AACAACTTTA 2860
Consensus GCATCATCCT CTGCATGGTC AGGTCATGGA TGAGCAGACG ATGGTGCAGG ATATCCTGCT NATGAAGCAG AACAACTTTA
Conservation
lac_operon_O157:-1 ACGCCGTGCG CTGTTCGCAT TATCCGAACC ATCCGCTGTG GTACACCCTG TGCGACCGCT ACGGCCTGTA TGTGGTGGAT 2940
lac_operon_MG165-1 ACGCCGTGCG CTGTTCGCAT TATCCGAACC ATCCGCTGTG GTACACGCTG TGCGACCGCT ACGGCCTGTA TGTGGTGGAT 2940
Consensus ACGCCGTGCG CTGTTCGCAT TATCCGAACC ATCCGCTGTG GTACACNCTG TGCGACCGCT ACGGCCTGTA TGTGGTGGAT
Conservation
lac_operon_O157:-1 GAAGCCAATA TTGAAACCCA CGGCATGGTG CCAATGAATC GTCTGACCGA TGATCCGCGC TGGTTACCGG CCATGAGCGA 3020
lac_operon_MG165-1 GAAGCCAATA TTGAAACCCA CGGCATGGTG CCAATGAATC GTCTGACCGA TGATCCGCGC TGGCTACCGG CGATGAGCGA 3020
Consensus GAAGCCAATA TTGAAACCCA CGGCATGGTG CCAATGAATC GTCTGACCGA TGATCCGCGC TGGNTACCGG CNATGAGCGA
Conservation
lac_operon_O157:-1 ACGAGTAACA CGAATGGTAC AGCGCGATCG TAATCACCCG AGTGTGATCA TCTGGTCGCT GGGGAATGAG TCAGGCCACG 3100
lac_operon_MG165-1 ACGCGTAACG CGAATGGTGC AGCGCGATCG TAATCACCCG AGTGTGATCA TCTGGTCGCT GGGGAATGAA TCAGGCCACG 3100
Consensus ACGNGTAACN CGAATGGTNC AGCGCGATCG TAATCACCCG AGTGTGATCA TCTGGTCGCT GGGGAATGAN TCAGGCCACG
Conservation

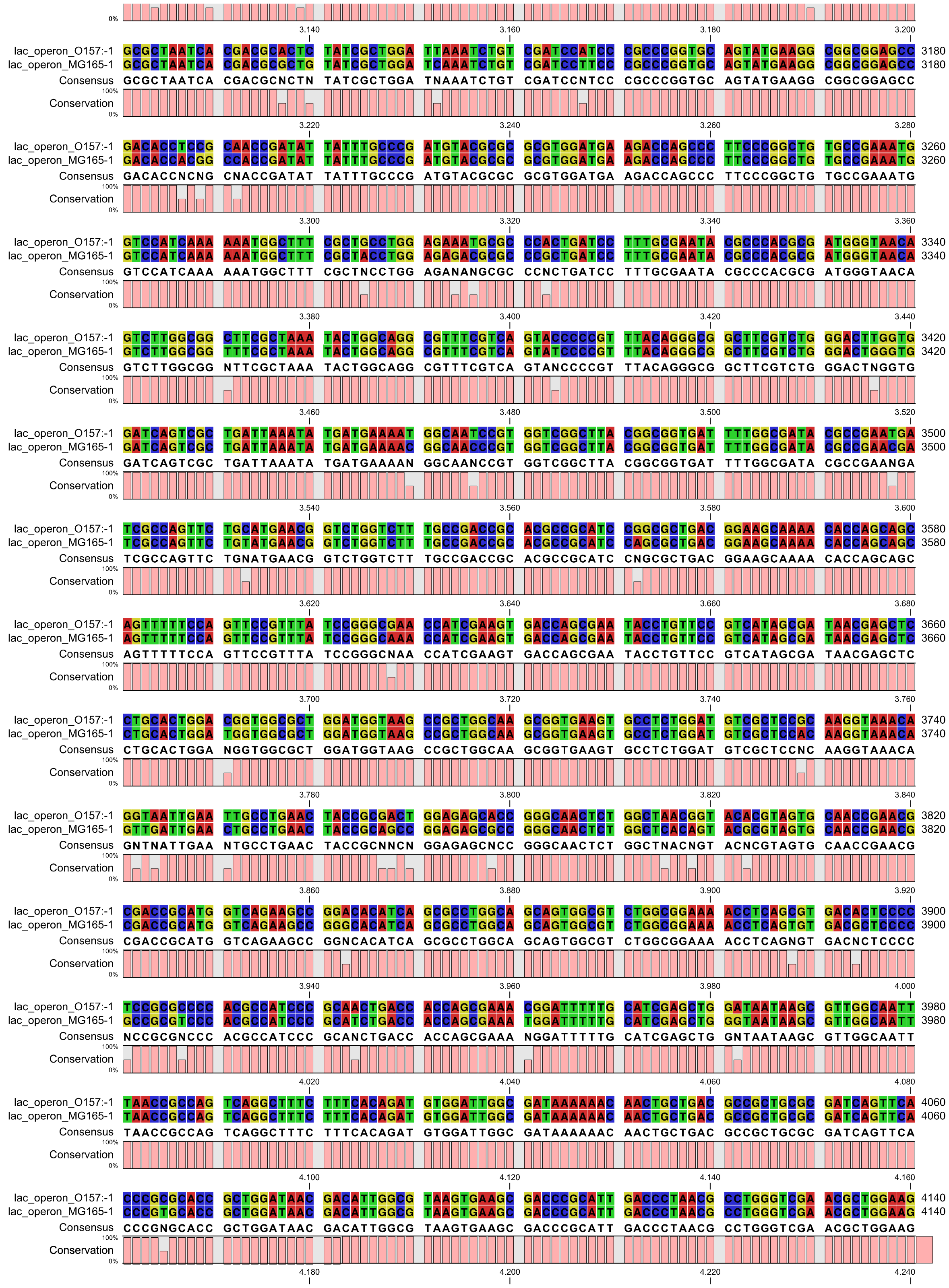
0%
3.140 3.160 3.180 3.200
lac_operon_O157:-1 GCGCTAATCA CGACGCACTC TATCGCTGGA TTAAATCTGT CGATCCATCC CGCCCGGTGC AGTATGAAGG CGGCGGAGCC 3180
lac_operon_MG165-1 GCGCTAATCA CGACGCGCTG TATCGCTGGA TCAAATCTGT CGATCCTTCC CGCCCGGTGC AGTATGAAGG CGGCGGAGCC 3180
Consensus GCGCTAATCA CGACGCNCTN TATCGCTGGA TNAAATCTGT CGATCCNTCC CGCCCGGTGC AGTATGAAGG CGGCGGAGCC
Conservation 100% 0%
3.220 3.240 3.260 3.280
lac_operon_O157:-1 GACACCTCCG CAACCGATAT TATTTGCCCG ATGTACGCGC GCGTGGATGA AGACCAGCCC TTCCCGGCTG TGCCGAAATG 3260
lac_operon_MG165-1 GACACCAGGG CCACCGATAT TATTTGCCCG ATGTACGCGC GCGTGGATGA AGACCAGCCC TTCCCGGCTG TGCCGAAATG 3260
Consensus GACACCNCNG CNACCGATAT TATTTGCCCG ATGTACGCGC GCGTGGATGA AGACCAGCCC TTCCCGGCTG TGCCGAAATG
Conservation 100% 0%
3.300 3.320 3.340 3.360
lac_operon_O157:-1 GTCCATCAAA AAATGGCTTT CGCTGCCTGG AGAAATGCGC CCACTGATCC TTTGCGAATA CGCCCACGCG ATGGGTAACA 3340
lac_operon_MG165-1 GTCCATCAAA AAATGGCTTT CGCTACCTGG AGAGACGCGC CCGCTGATCC TTTGCGAATA CGCCCACGCG ATGGGTAACA 3340
Consensus GTCCATCAAA AAATGGCTTT CGCTNCCTGG AGANANGCGC CCNCTGATCC TTTGCGAATA CGCCCACGCG ATGGGTAACA
Conservation 100% 0%
3.380 3.400 3.420 3.440
lac_operon_O157:-1 GTCTTGGCGG CTTCGCTAAA TACTGGCAGG CGTTTCGTCA GTACCCCCGT TTACAGGGCG GCTTCGTCTG GGACTTGGTG 3420
lac_operon_MG165-1 GTCTTGGCGG TTTCGCTAAA TACTGGCAGG CGTTTCGTCA GTATCCCCGT TTACAGGGCG GCTTCGTCTG GGACTGGGTG 3420
Consensus GTCTTGGCGG NTTCGCTAAA TACTGGCAGG CGTTTCGTCA GTANCCCCGT TTACAGGGCG GCTTCGTCTG GGACTNGGTG
Conservation 100% 0%
3.460 3.480 3.500 3.520
lac_operon_O157:-1 GATCAGTCGC TGATTAAATA TGATGAAAAT GGCAATCCGT GGTCGGCTTA CGGCGGTGAT TTTGGCGATA CGCCGAATGA 3500
lac_operon_MG165-1 GATCAGTCGC TGATTAAATA TGATGAAAAC GGCAACCCGT GGTCGGCTTA CGGCGGTGAT TTTGGCGATA CGCCGAACGA 3500
Consensus GATCAGTCGC TGATTAAATA TGATGAAAAN GGCAANCCGT GGTCGGCTTA CGGCGGTGAT TTTGGCGATA CGCCGAANGA
Conservation 100% 0%
3.540 3.560 3.580 3.600
lac_operon_O157:-1 TCGCCAGTTC TGCATGAACG GTCTGGTCTT TGCCGACCGC ACGCCGCATC CGGCGCTGAC GGAAGCAAAA CACCAGCAGC 3580
lac_operon_MG165-1 TCGCCAGTTC TGTATGAACG GTCTGGTCTT TGCCGACCGC ACGCCGCATC CAGCGCTGAC GGAAGCAAAA CACCAGCAGC 3580
Consensus TCGCCAGTTC TGNATGAACG GTCTGGTCTT TGCCGACCGC ACGCCGCATC CNGCGCTGAC GGAAGCAAAA CACCAGCAGC
Conservation 100% 0%
3.620 3.640 3.660 3.680
lac_operon_O157:-1 AGTTTTTCCA GTTCCGTTTA TCCGGGCGAA CCATCGAAGT GACCAGCGAA TACCTGTTCC GTCATAGCGA TAACGAGCTC 3660
lac_operon_MG165-1 AGTTTTTCCA GTTCCGTTTA TCCGGGCAAA CCATCGAAGT GACCAGCGAA TACCTGTTCC GTCATAGCGA TAACGAGCTC 3660
Consensus AGTTTTTCCA GTTCCGTTTA TCCGGGCNAA CCATCGAAGT GACCAGCGAA TACCTGTTCC GTCATAGCGA TAACGAGCTC
Conservation 100% 0%
3.700 3.720 3.740 3.760
lac_operon_O157:-1 CTGCACTGGA CGGTGGCGCT GGATGGTAAG CCGCTGGCAA GCGGTGAAGT GCCTCTGGAT GTCGCTCCGC AAGGTAAACA 3740
lac_operon_MG165-1 CTGCACTGGA TGGTGGCGCT GGATGGTAAG CCGCTGGCAA GCGGTGAAGT GCCTCTGGAT GTCGCTCCAC AAGGTAAACA 3740
Consensus CTGCACTGGA NGGTGGCGCT GGATGGTAAG CCGCTGGCAA GCGGTGAAGT GCCTCTGGAT GTCGCTCCNC AAGGTAAACA
Conservation 100% 0%
3.780 3.800 3.820 3.840
lac_operon_O157:-1 GGTAATTGAA TTGCCTGAAC TACCGCGACT GGAGAGCACC GGGCAACTCT GGCTAACGGT ACACGTAGTG CAACCGAACG 3820
lac_operon_MG165-1 GTTGATTGAA CTGCCTGAAC TACCGCAGCC GGAGAGCGCC GGGCAACTCT GGCTCACAGT ACGCGTAGTG CAACCGAACG 3820
Consensus GNTNATTGAA NTGCCTGAAC TACCGCNNCN GGAGAGCNCC GGGCAACTCT GGCTNACNGT ACNCGTAGTG CAACCGAACG
Conservation 100% 0%
3.860 3.880 3.900 3.920
lac_operon_O157:-1 CGACCGCATG GTCAGAAGCC GGACACATCA GCGCCTGGCA GCAGTGGCGT CTGGCGGAAA ACCTCAGCGT GACACTCCCC 3900
lac_operon_MG165-1 CGACCGCATG GTCAGAAGCC GGGCACATCA GCGCCTGGCA GCAGTGGCGT CTGGCGGAAA ACCTCAGTGT GACGCTCCCC 3900
Consensus CGACCGCATG GTCAGAAGCC GGNCACATCA GCGCCTGGCA GCAGTGGCGT CTGGCGGAAA ACCTCAGNGT GACNCTCCCC
Conservation 100% 0%
3.940 3.960 3.980 4.000
lac_operon_O157:-1 TCCGCGCCCC ACGCCATCCC GCAACTGACC ACCAGCGAAA CGGATTTTTG CATCGAGCTG GATAATAAGC GTTGGCAATT 3980
lac_operon_MG165-1 GCCGCGTCCC ACGCCATCCC GCATCTGACC ACCAGCGAAA TGGATTTTTG CATCGAGCTG GGTAATAAGC GTTGGCAATT 3980
Consensus NCCGCGNCCC ACGCCATCCC GCANCTGACC ACCAGCGAAA NGGATTTTTG CATCGAGCTG GNTAATAAGC GTTGGCAATT
Conservation 100% 0%
4.020 4.040 4.060 4.080
lac_operon_O157:-1 TAACCGCCAG TCAGGCTTTC TTTCACAGAT GTGGATTGGC GATAAAAAAC AACTGCTGAC GCCGCTGCGC GATCAGTTCA 4060
lac_operon_MG165-1 TAACCGCCAG TCAGGCTTTC TTTCACAGAT GTGGATTGGC GATAAAAAAC AACTGCTGAC GCCGCTGCGC GATCAGTTCA 4060
Consensus TAACCGCCAG TCAGGCTTTC TTTCACAGAT GTGGATTGGC GATAAAAAAC AACTGCTGAC GCCGCTGCGC GATCAGTTCA
Conservation 100% 0%
4.100 4.120 4.140 4.160
lac_operon_O157:-1 CCCGCGCACC GCTGGATAAC GACATTGGCG TAAGTGAAGC GACCCGCATT GACCCTAACG CCTGGGTCGA ACGCTGGAAG 4140
lac_operon_MG165-1 CCCGTGCACC GCTGGATAAC GACATTGGCG TAAGTGAAGC GACCCGCATT GACCCTAACG CCTGGGTCGA ACGCTGGAAG 4140
Consensus CCCGNGCACC GCTGGATAAC GACATTGGCG TAAGTGAAGC GACCCGCATT GACCCTAACG CCTGGGTCGA ACGCTGGAAG
Conservation 100% 0%
4.180 4.200 4.220 4.240

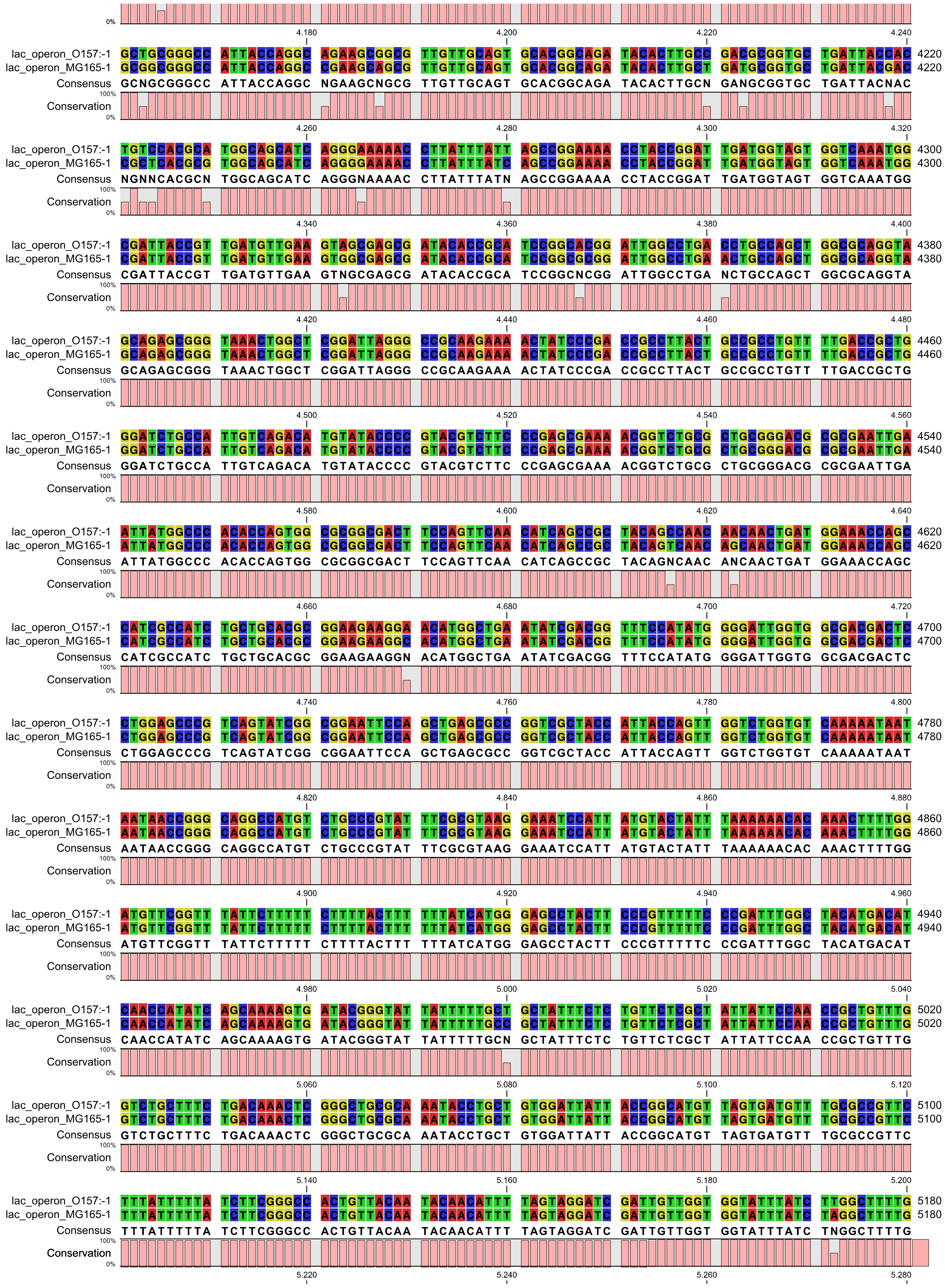

lac_operon_O157:-1 GCTGCGGGCC ATTACCAGGC AGAAGCGGCG TTGTTGCAGT GCACGGCAGA TACACTTGCC GACGCGGTGC TGATTACCAC 4220
lac_operon_MG165-1 GCGGCGGGCC ATTACCAGGC CGAAGCAGCG TTGTTGCAGT GCACGGCAGA TACACTTGCT GATGCGGTGC TGATTACGAC 4220
Consensus GCNGCGGGCC ATTACCAGGC NGAAGCNGCG TTGTTGCAGT GCACGGCAGA TACACTTGCN GANGCGGTGC TGATTACNAC
Conservation
lac_operon_O157:-1 TGTCCACGCA TGGCAGCATC AGGGAAAAAC CTTATTTATT AGCCGGAAAA CCTACCGGAT TGATGGTAGT GGTCAAATGG 4300
lac_operon_MG165-1 CGCTCACGCG TGGCAGCATC AGGGGAAAAC CTTATTTATC AGCCGGAAAA CCTACCGGAT TGATGGTAGT GGTCAAATGG 4300
Consensus NGNNCACGCN TGGCAGCATC AGGGNAAAAC CTTATTTATN AGCCGGAAAA CCTACCGGAT TGATGGTAGT GGTCAAATGG
Conservation
lac_operon_O157:-1 CGATTACCGT TGATGTTGAA GTAGCGAGCG ATACACCGCA TCCGGCACGG ATTGGCCTGA CCTGCCAGCT GGCGCAGGTA 4380
lac_operon_MG165-1 CGATTACCGT TGATGTTGAA GTGGCGAGCG ATACACCGCA TCCGGCGCGG ATTGGCCTGA ACTGCCAGCT GGCGCAGGTA 4380
Consensus CGATTACCGT TGATGTTGAA GTNGCGAGCG ATACACCGCA TCCGGCNCGG ATTGGCCTGA NCTGCCAGCT GGCGCAGGTA
Conservation
lac_operon_O157:-1 GCAGAGCGGG TAAACTGGCT CGGATTAGGG CCGCAAGAAA ACTATCCCGA CCGCCTTACT GCCGCCTGTT TTGACCGCTG 4460
lac_operon_MG165-1 GCAGAGCGGG TAAACTGGCT CGGATTAGGG CCGCAAGAAA ACTATCCCGA CCGCCTTACT GCCGCCTGTT TTGACCGCTG 4460
Consensus GCAGAGCGGG TAAACTGGCT CGGATTAGGG CCGCAAGAAA ACTATCCCGA CCGCCTTACT GCCGCCTGTT TTGACCGCTG
Conservation
lac_operon_O157:-1 GGATCTGCCA TTGTCAGACA TGTATACCCC GTACGTCTTC CCGAGCGAAA ACGGTCTGCG CTGCGGGACG CGCGAATTGA 4540
lac_operon_MG165-1 GGATCTGCCA TTGTCAGACA TGTATACCCC GTACGTCTTC CCGAGCGAAA ACGGTCTGCG CTGCGGGACG CGCGAATTGA 4540
Consensus GGATCTGCCA TTGTCAGACA TGTATACCCC GTACGTCTTC CCGAGCGAAA ACGGTCTGCG CTGCGGGACG CGCGAATTGA
Conservation
lac_operon_O157:-1 ATTATGGCCC ACACCAGTGG CGCGGCGACT TCCAGTTCAA CATCAGCCGC TACAGCCAAC AACAACTGAT GGAAACCAGC 4620
lac_operon_MG165-1 ATTATGGCCC ACACCAGTGG CGCGGCGACT TCCAGTTCAA CATCAGCCGC TACAGTCAAC AGCAACTGAT GGAAACCAGC 4620
Consensus ATTATGGCCC ACACCAGTGG CGCGGCGACT TCCAGTTCAA CATCAGCCGC TACAGNCAAC ANCAACTGAT GGAAACCAGC
Conservation
lac_operon_O157:-1 CATCGCCATC TGCTGCACGC GGAAGAAGGA ACATGGCTGA ATATCGACGG TTTCCATATG GGGATTGGTG GCGACGACTC 4700
lac_operon_MG165-1 CATCGCCATC TGCTGCACGC GGAAGAAGGC ACATGGCTGA ATATCGACGG TTTCCATATG GGGATTGGTG GCGACGACTC 4700
Consensus CATCGCCATC TGCTGCACGC GGAAGAAGGN ACATGGCTGA ATATCGACGG TTTCCATATG GGGATTGGTG GCGACGACTC
Conservation
lac_operon_O157:-1 CTGGAGCCCG TCAGTATCGG CGGAATTCCA GCTGAGCGCC GGTCGCTACC ATTACCAGTT GGTCTGGTGT CAAAAATAAT 4780
lac_operon_MG165-1 CTGGAGCCCG TCAGTATCGG CGGAATTCCA GCTGAGCGCC GGTCGCTACC ATTACCAGTT GGTCTGGTGT CAAAAATAAT 4780
Consensus CTGGAGCCCG TCAGTATCGG CGGAATTCCA GCTGAGCGCC GGTCGCTACC ATTACCAGTT GGTCTGGTGT CAAAAATAAT
Conservation
lac_operon_O157:-1 AATAACCGGG CAGGCCATGT CTGCCCGTAT TTCGCGTAAG GAAATCCATT ATGTACTATT TAAAAAACAC AAACTTTTGG 4860
lac_operon_MG165-1 AATAACCGGG CAGGCCATGT CTGCCCGTAT TTCGCGTAAG GAAATCCATT ATGTACTATT TAAAAAACAC AAACTTTTGG 4860
Consensus AATAACCGGG CAGGCCATGT CTGCCCGTAT TTCGCGTAAG GAAATCCATT ATGTACTATT TAAAAAACAC AAACTTTTGG
Conservation
lac_operon_O157:-1 ATGTTCGGTT TATTCTTTTT CTTTTACTTT TTTATCATGG GAGCCTACTT CCCGTTTTTC CCGATTTGGC TACATGACAT 4940
lac_operon_MG165-1 ATGTTCGGTT TATTCTTTTT CTTTTACTTT TTTATCATGG GAGCCTACTT CCCGTTTTTC CCGATTTGGC TACATGACAT 4940
Consensus ATGTTCGGTT TATTCTTTTT CTTTTACTTT TTTATCATGG GAGCCTACTT CCCGTTTTTC CCGATTTGGC TACATGACAT
Conservation
lac_operon_O157:-1 CAACCATATC AGCAAAAGTG ATACGGGTAT TATTTTTGCT GCTATTTCTC TGTTCTCGCT ATTATTCCAA CCGCTGTTTG 5020
lac_operon_MG165-1 CAACCATATC AGCAAAAGTG ATACGGGTAT TATTTTTGCC GCTATTTCTC TGTTCTCGCT ATTATTCCAA CCGCTGTTTG 5020
Consensus CAACCATATC AGCAAAAGTG ATACGGGTAT TATTTTTGCN GCTATTTCTC TGTTCTCGCT ATTATTCCAA CCGCTGTTTG
Conservation
lac_operon_O157:-1 GTCTGCTTTC TGACAAACTC GGGCTGCGCA AATACCTGCT GTGGATTATT ACCGGCATGT TAGTGATGTT TGCGCCGTTC 5100
lac_operon_MG165-1 GTCTGCTTTC TGACAAACTC GGGCTGCGCA AATACCTGCT GTGGATTATT ACCGGCATGT TAGTGATGTT TGCGCCGTTC 5100
Consensus GTCTGCTTTC TGACAAACTC GGGCTGCGCA AATACCTGCT GTGGATTATT ACCGGCATGT TAGTGATGTT TGCGCCGTTC
Conservation
lac_operon_O157:-1 TTTATTTTTA TCTTCGGGCC ACTGTTACAA TACAACATTT TAGTAGGATC GATTGTTGGT GGTATTTATC TTGGCTTTTG 5180
lac_operon_MG165-1 TTTATTTTTA TCTTCGGGCC ACTGTTACAA TACAACATTT TAGTAGGATC GATTGTTGGT GGTATTTATC TAGGCTTTTG 5180
Consensus TTTATTTTTA TCTTCGGGCC ACTGTTACAA TACAACATTT TAGTAGGATC GATTGTTGGT GGTATTTATC TNGGCTTTTG
Conservation

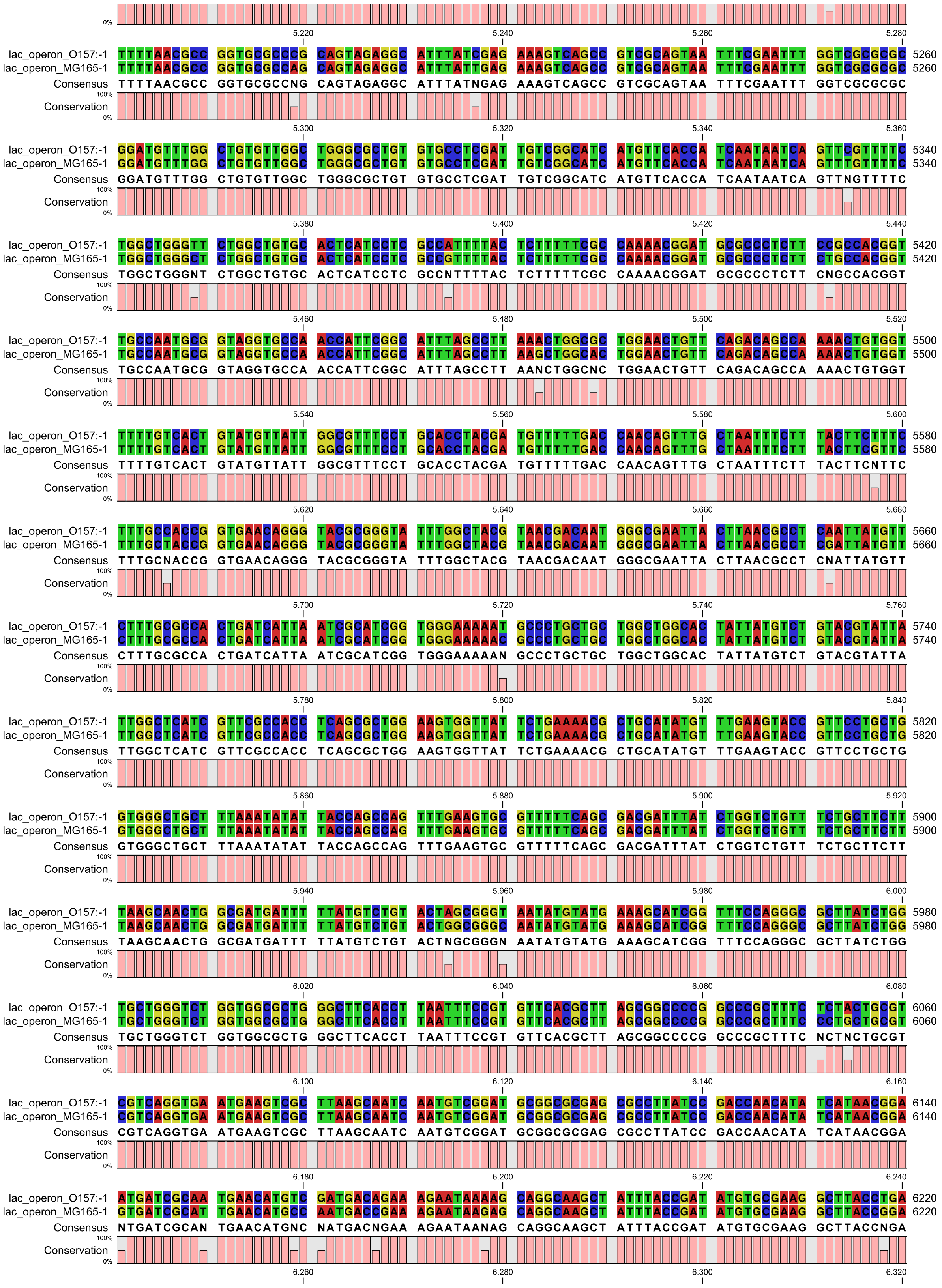

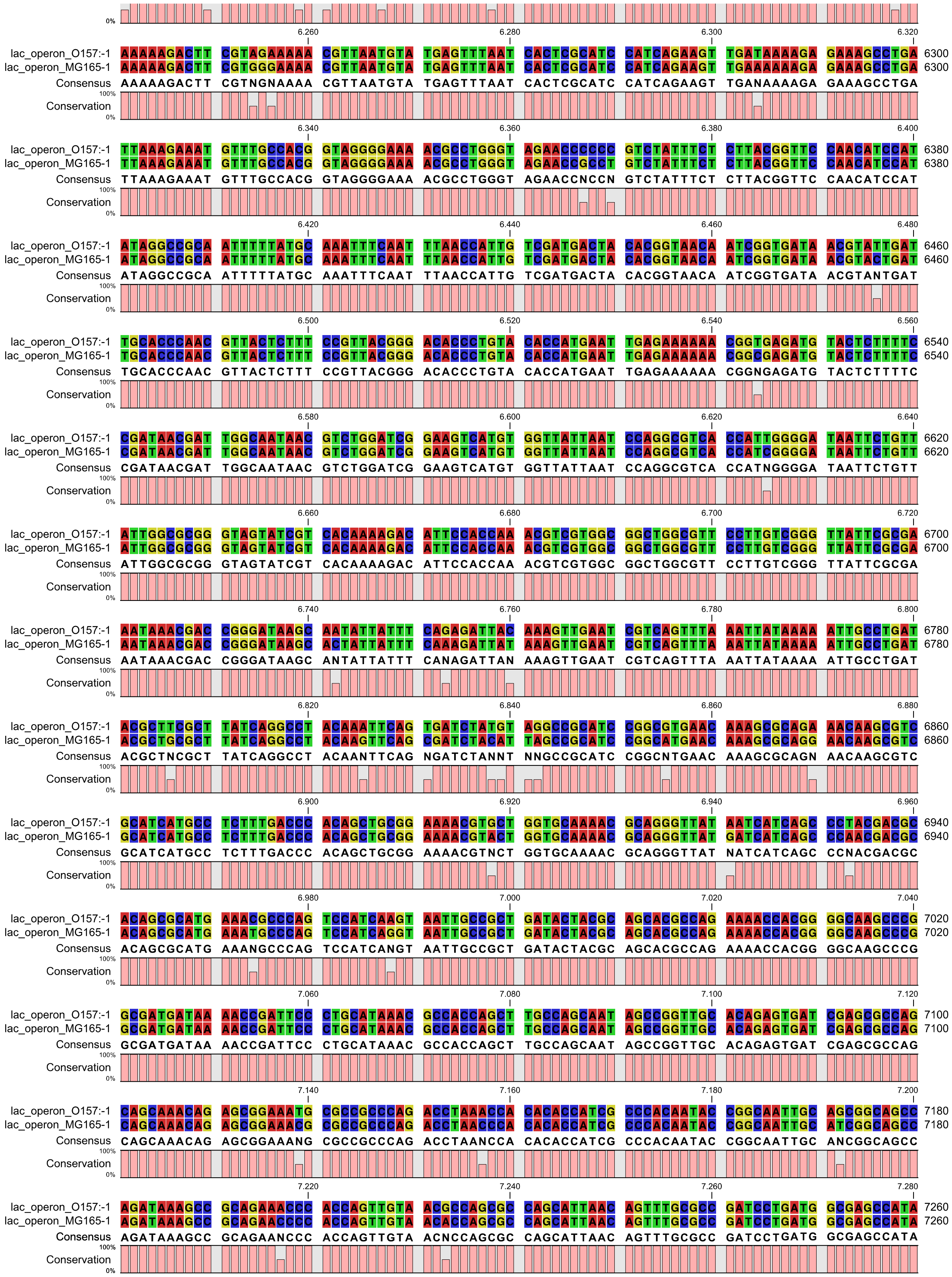
lac_operon_O157:-1
lac_operon_MG165-1
Consensus
Conservation
100%
0%

A

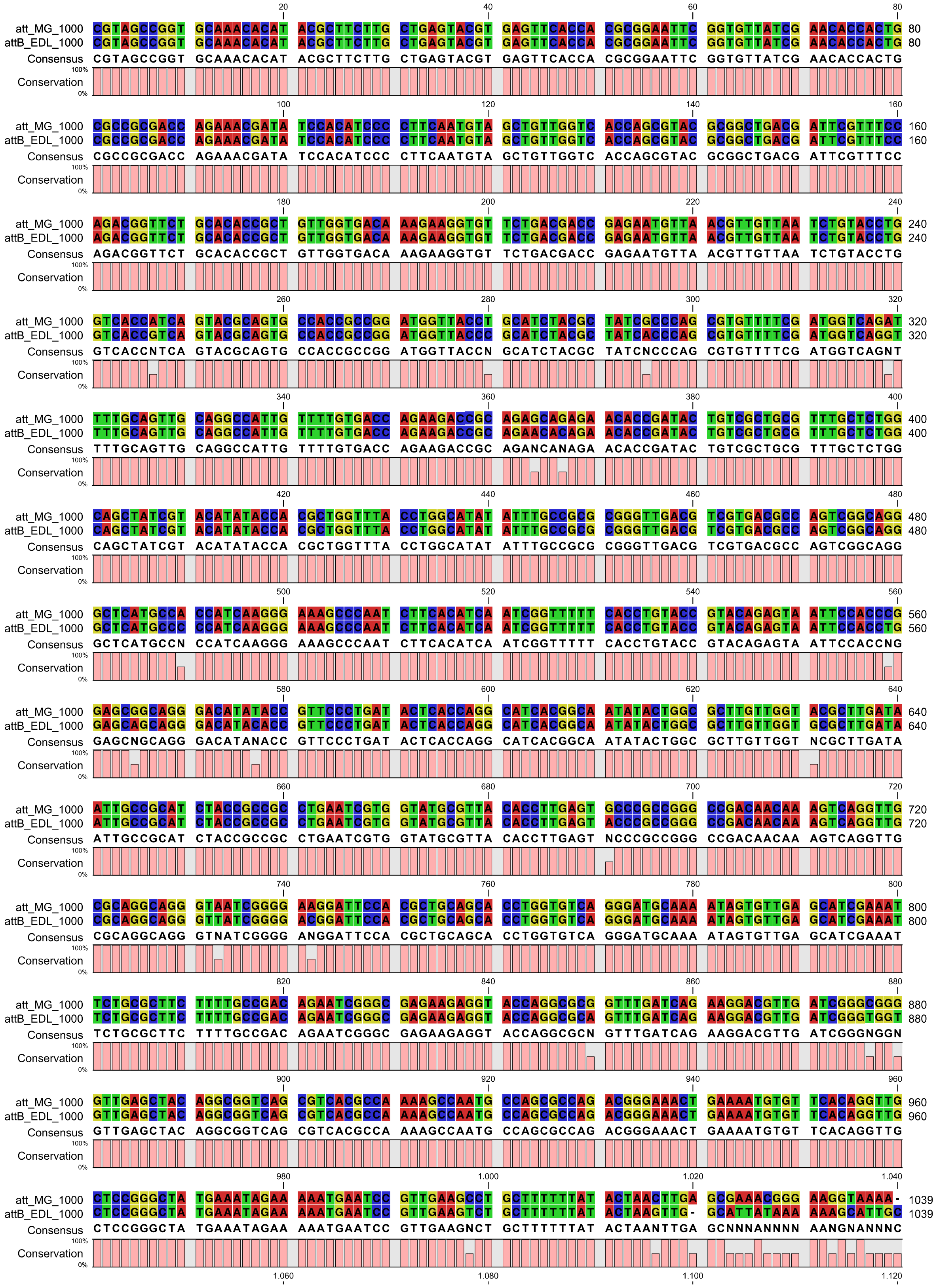

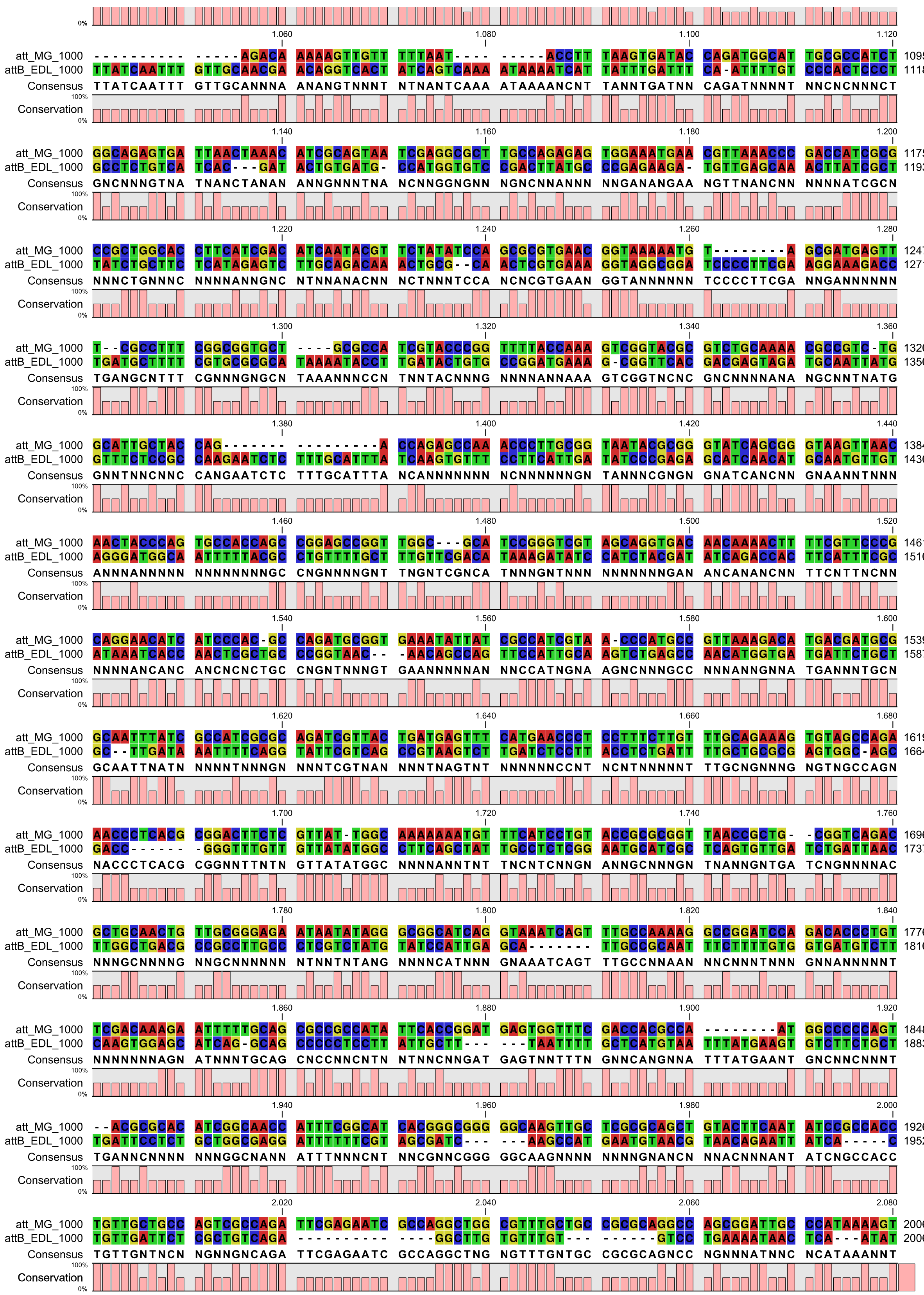

B

Supplementary Figure S1. (**A**) Shown is an alignment of the nucleotide sequences of the regulatory gene *lacI* and the metabolic operon *lacZYA* (-/+ 499 bp) of EHEC O157:H7 EDL933 and *E. coli* K-12 MG1655. (**B**) Shown is an alignment of the nucleotide sequences of the conserved 15 bp phage

lambda attachment site (*attB*) and the DNA sequences 1000 bp upstream and downstream of *attB* of EHEC O157:H7 EDL933 and *E. coli* K-12 MG1655.

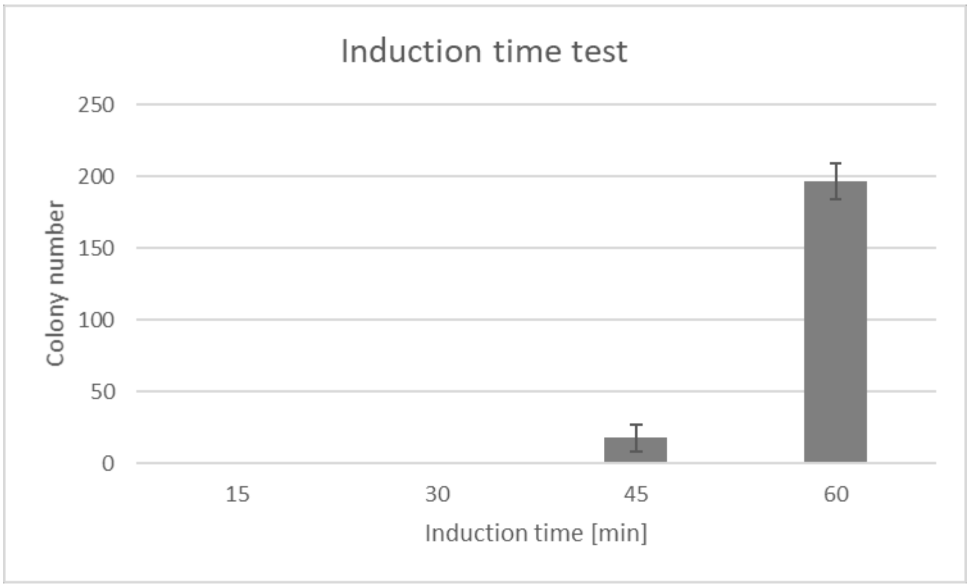

Supplementary Figure S2. Induction time test. Shown is the average number of colonies obtained with 1 ng of recombination substrate for the *lacZYA* locus when using pKD46 in *E. coli* K-12 MG1655. Shown are the average colony numbers and the standard deviations from three technical replicates for each time point.

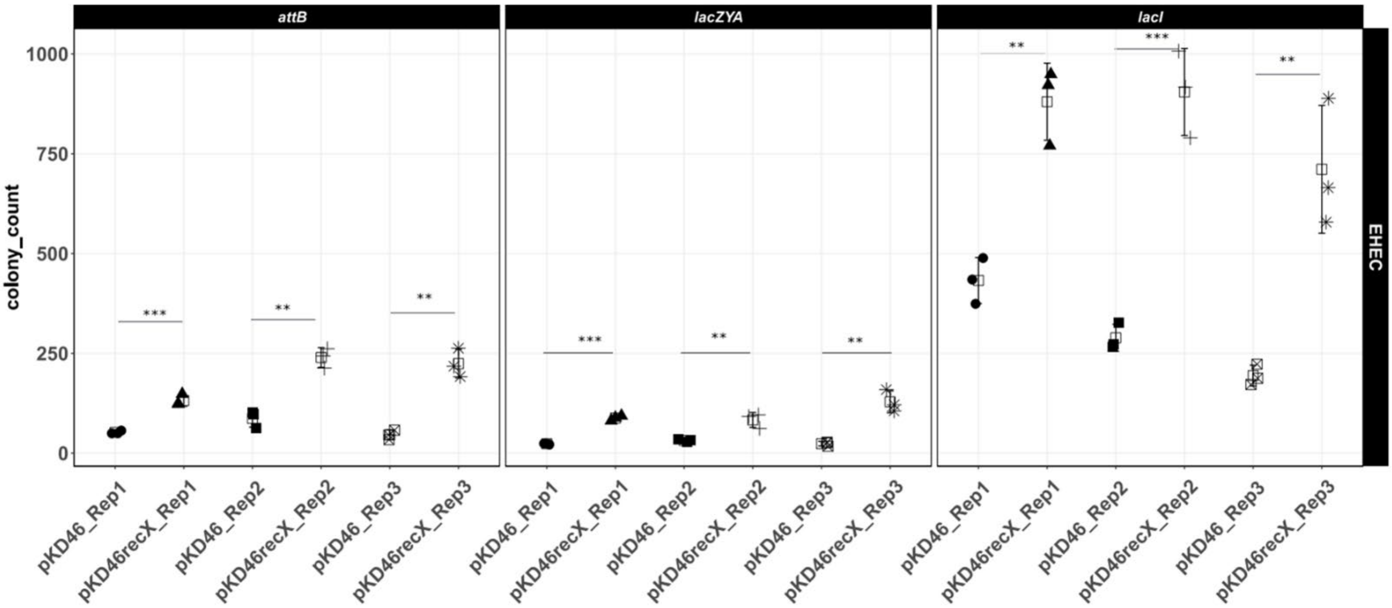

Supplementary Figure S3. Numbers of chloramphenicol-resistant colonies obtained for the indicated genetic locus for EHEC O157:H7 EDL933 *Δstx1/2*. Shown are the numbers and standard deviations of colonies obtained from each of the three technical replicates in each experiment. Statistically significant t test comparisons are indicated by * ($p < 0.05$), ** ($p<0.01$), *** ($p < 0.001$) and "ns" if not statistically significant.

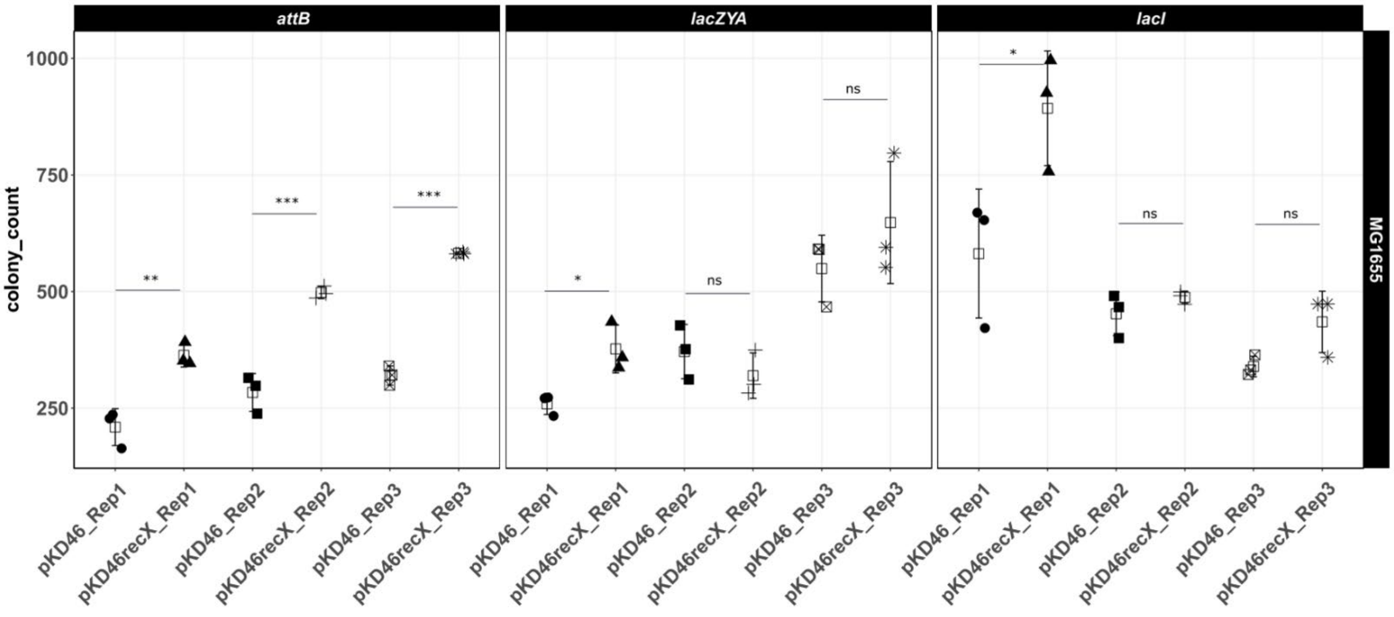

Supplementary Figure S4. Numbers of chloramphenicol-resistant colonies obtained for the indicated genetic locus for *E. coli* K-12 MG1655. Shown are the numbers and standard deviations of colonies obtained from each of the three technical replicates in each experiment. Statistically significant t test comparisons are indicated by * ($p < 0.05$), ** ($p<0.01$), *** ($p < 0.001$) and "ns" if not statistically significant.

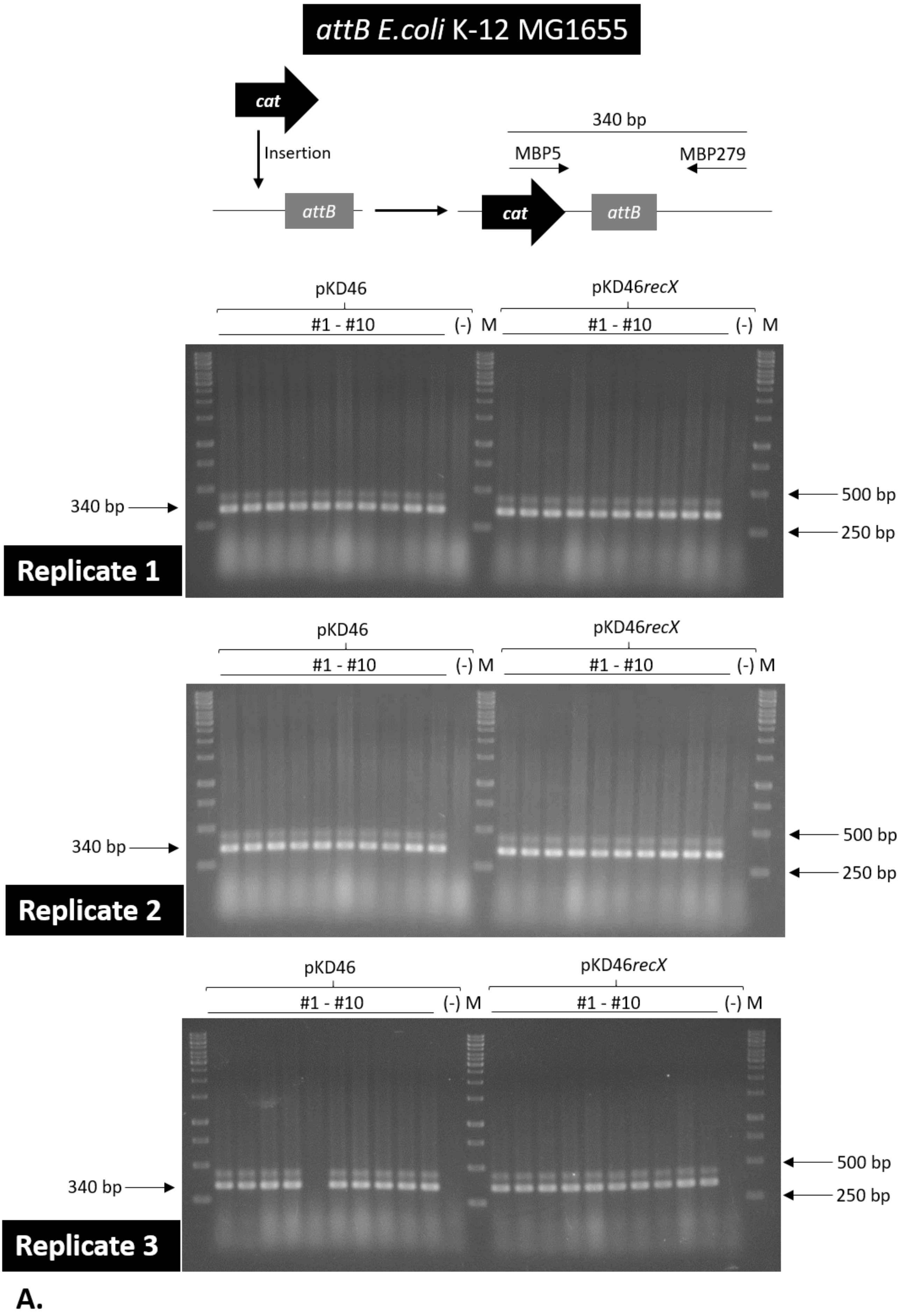

attB E.coli K-12 MG1655
cat
Insertion
attB
340 bp
MBP5
MBP279
cat
attB
pKD46
pKD46recX
#1 - #10
(-) M
340 bp
500 bp
250 bp
Replicate 1
Replicate 2
Replicate 3
A.

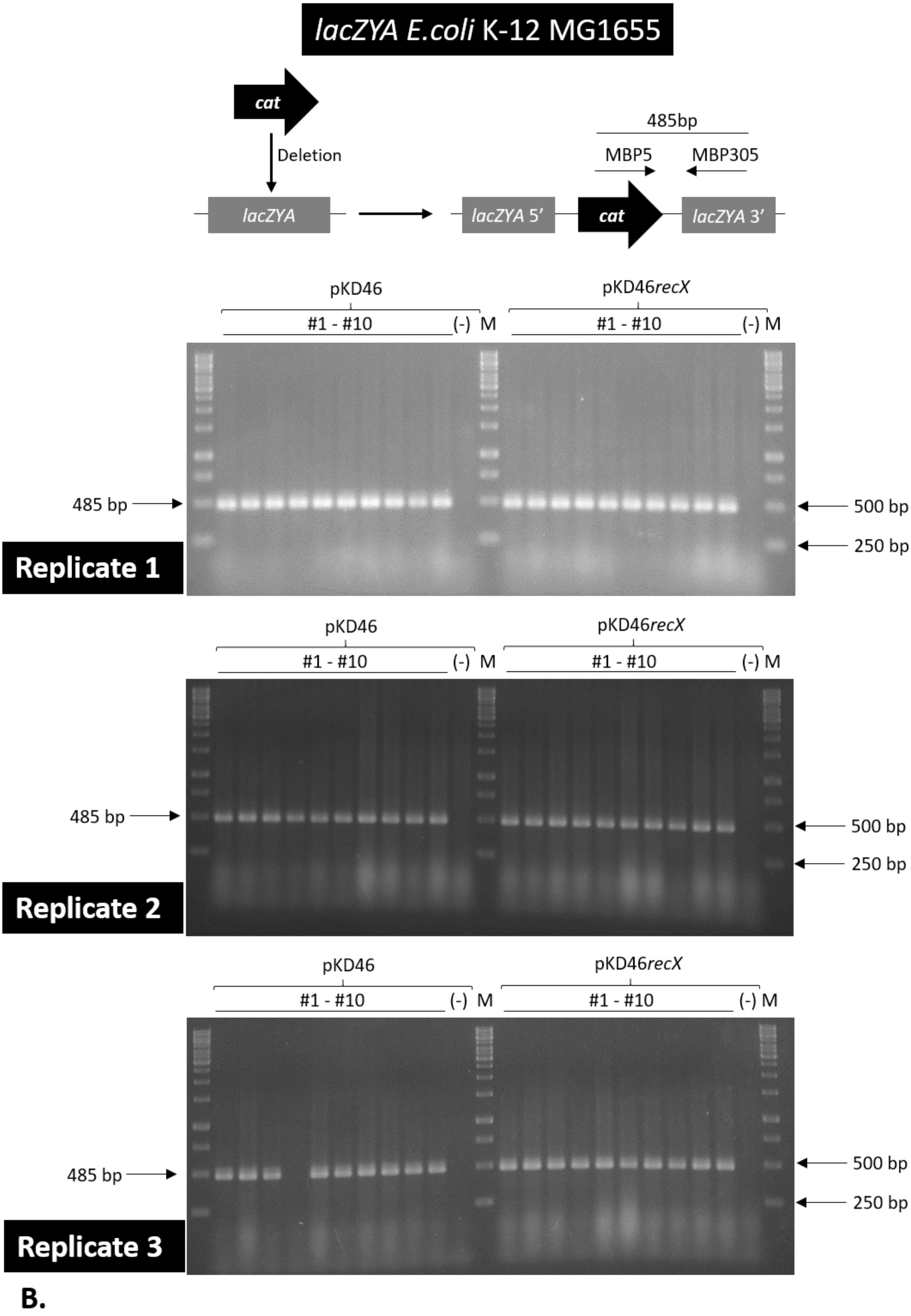
lacZYA E.coli K-12 MG1655
cat
Deletion
485bp
MBP5
MBP305
lacZYA
lacZYA 5'
cat
lacZYA 3'
pKD46
pKD46recX
#1 - #10
(-) M
485 bp
500 bp
250 bp
Replicate 1
Replicate 2
Replicate 3
B.

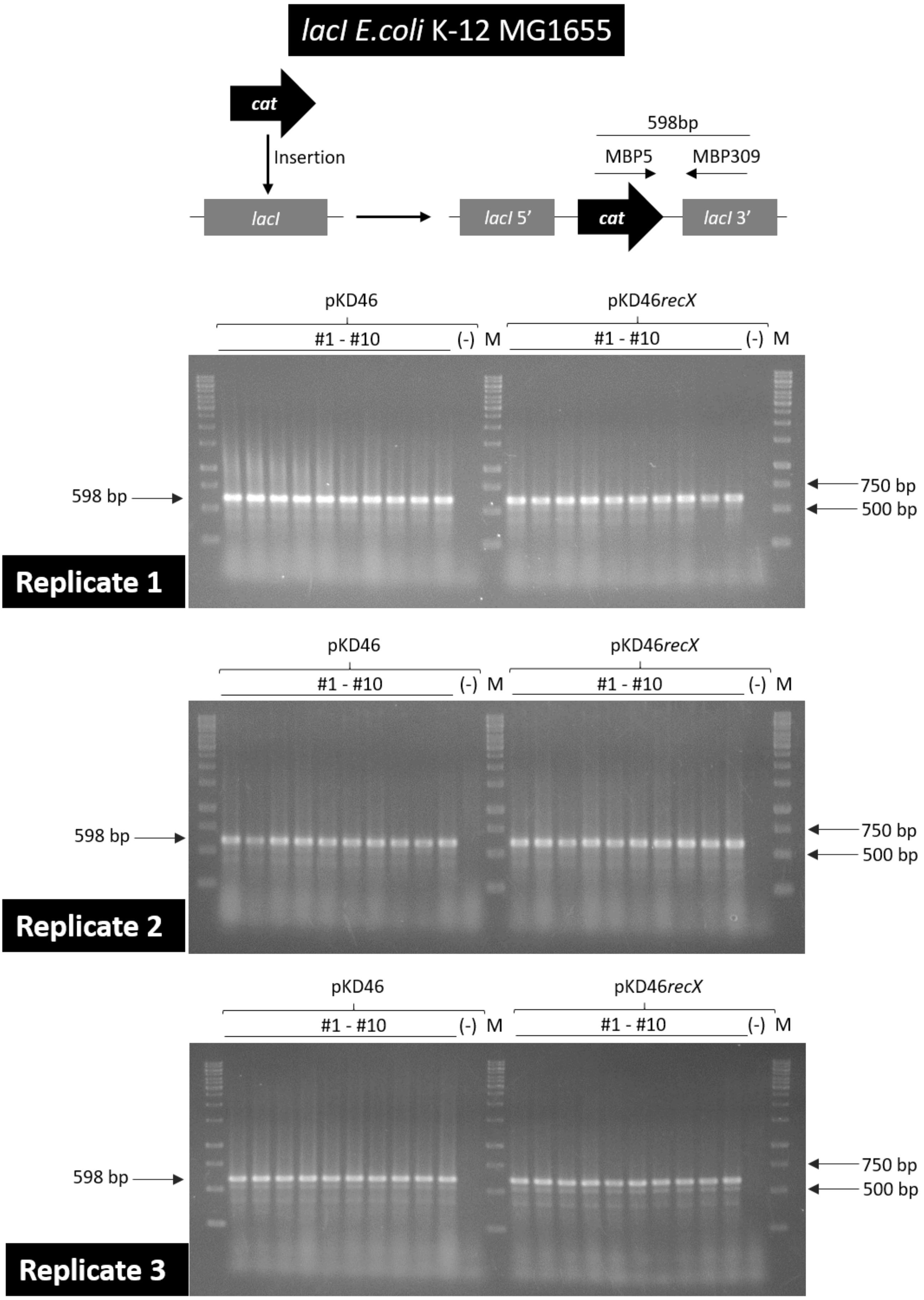
lacI E.coli K-12 MG1655
cat
Insertion
598bp
MBP5
MBP309
lacI
lacI 5'
cat
lacI 3'
pKD46
pKD46*recX*
#1 - #10
(-)
M
598 bp
750 bp
500 bp
Replicate 1
Replicate 2
Replicate 3

C.

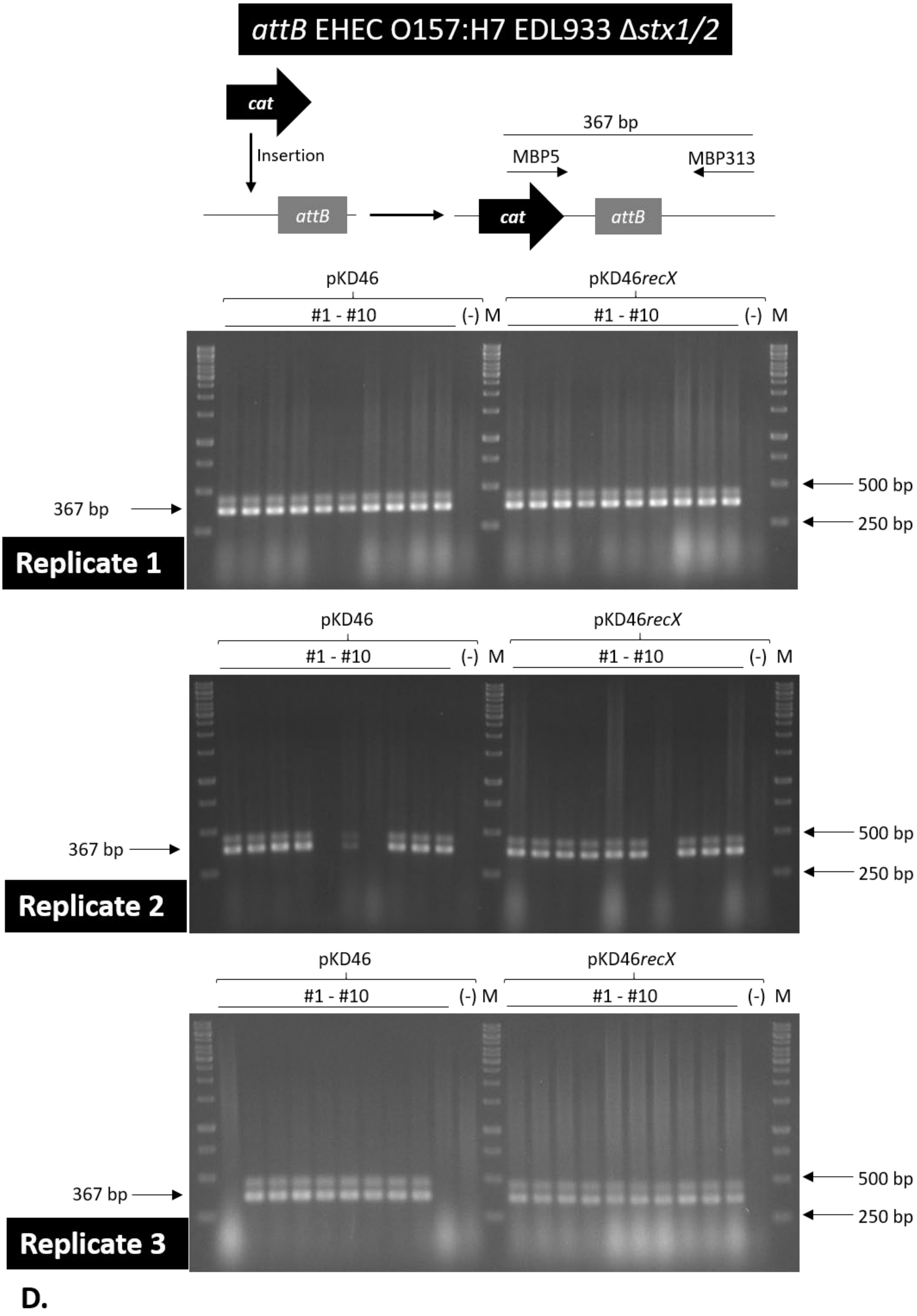
attB EHEC O157:H7 EDL933 Δstx1/2
cat
Insertion
attB
367 bp
MBP5
MBP313
cat
attB
pKD46
pKD46recX
#1 - #10
(-) M
#1 - #10
(-) M
367 bp
500 bp
250 bp
Replicate 1
pKD46
pKD46recX
#1 - #10
(-) M
#1 - #10
(-) M
367 bp
500 bp
250 bp
Replicate 2
pKD46
pKD46recX
#1 - #10
(-) M
#1 - #10
(-) M
367 bp
500 bp
250 bp
Replicate 3
D.

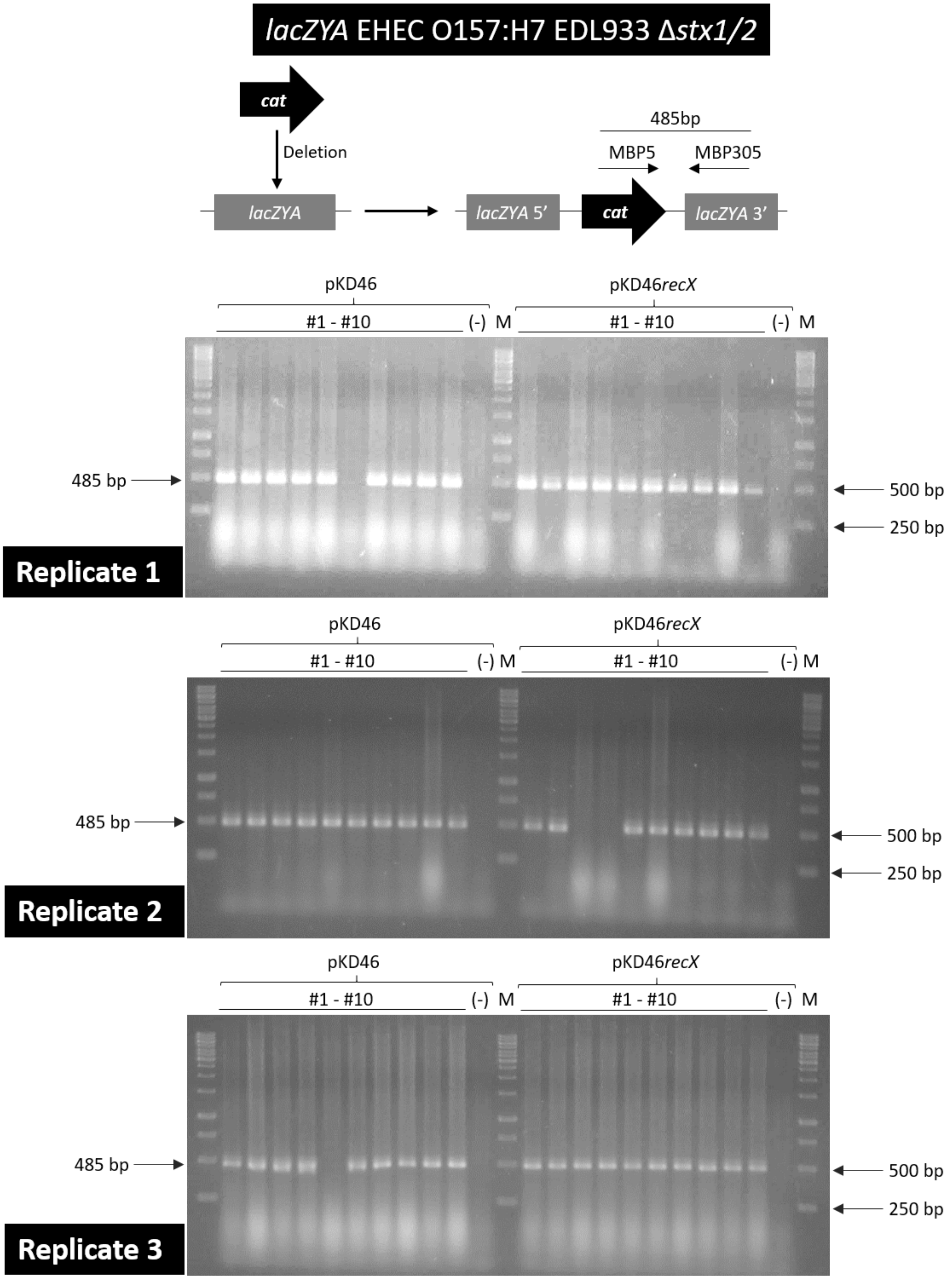

lacZYA EHEC O157:H7 EDL933 Δstx1/2
cat
Deletion
485bp
MBP5
MBP305
lacZYA
lacZYA 5'
cat
lacZYA 3'
pKD46
#1 - #10
(-) M
pKD46recX
#1 - #10
(-) M
485 bp
500 bp
250 bp
Replicate 1
pKD46
#1 - #10
(-) M
pKD46recX
#1 - #10
(-) M
485 bp
500 bp
250 bp
Replicate 2
pKD46
#1 - #10
(-) M
pKD46recX
#1 - #10
(-) M
485 bp
500 bp
250 bp
Replicate 3

E.

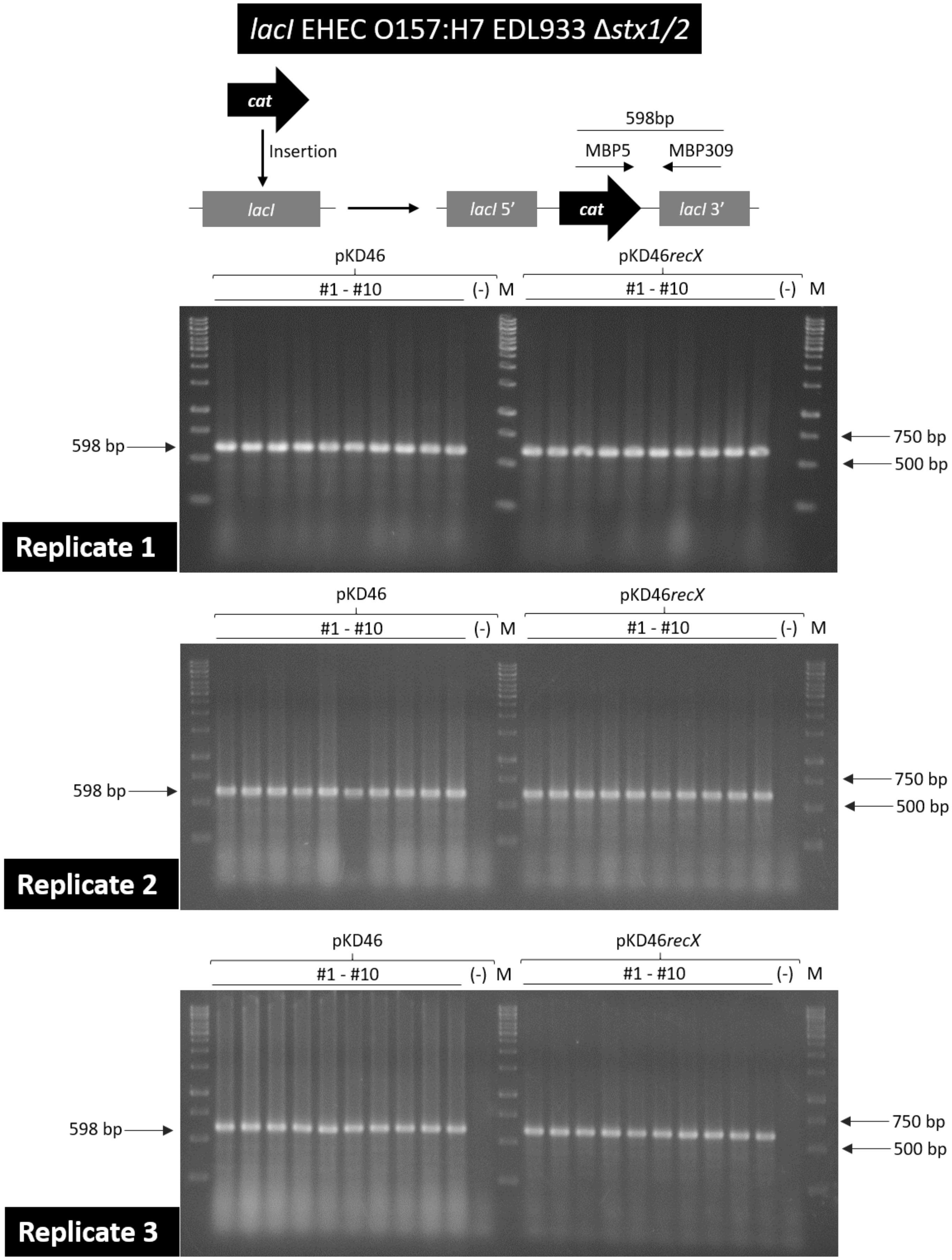
lacI EHEC O157:H7 EDL933 Δstx1/2
cat
Insertion
598bp
MBP5
MBP309
lacI
lacI 5'
cat
lacI 3'
pKD46
pKD46recX
#1 - #10
(-)
M
598 bp
750 bp
500 bp
Replicate 1
Replicate 2
Replicate 3

F.

Supplementary Figure S5. PCR analysis. Shown are the results of the colony PCR for each experiment. 10 randomly picked colonies per plasmid and biological replicate were analyzed by PCR using the primers that are indicated graphically on top of the images. Indicated are the numbers of colonies analyzed (#1 - #10) and the negative control (-) that was the same strain containing either pKD46 or pKD46*recX*. **A.** *attB* locus insertion in *E. coli* K-12 MG1655. **B.** *lacZYA* deletion in *E. coli* K-12 MG1655 **C.** *lacI* deletion in *E. coli* K-12 MG1655. **D.** *attB* locus insertion in EHEC O157:H7 EDL933 *Δstx1/2* **E.** *lacZYA* deletion in EHEC O157:H7 EDL933 *Δstx1/2* **F.** *lacI* deletion in EHEC O157:H7 EDL933 *Δstx1/2*. The vast majority of chloramphenicol-resistant colonies was PCR-positive. There was no difference in PCR-negative colonies between pKD46 and pKD46recX containing strains. For the details, see the text.

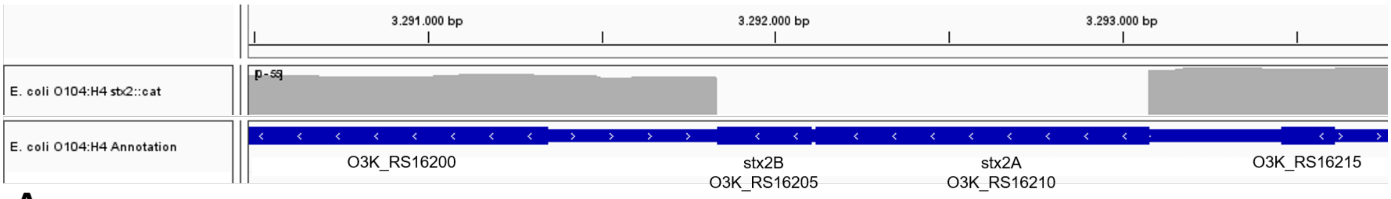

A.

…GAGTCCTCGATGGCGGTCCATTATCTGCATTATGCGTTGTTAGCTCAGCCGGACAGAGCAATTGCCTTCTGAGCAA
TCGGTCACTGGTTCGAATCCAGTACAACGCGCCATATTTATTTACCAGGCTCGCTTTTGCGGGCCTTTTTTATATCT
GCGCCGGGTCTGGTGCTGATTACTTCAGCCAAAAGGAACACCTGTAT**ACGACGTTGTAAAACGACGGCCAGTGCCAA**
GCTTGCATGCCTGCAGCGCCGAATAAATACCTGTGACGGAAGATCACTTCGCAGAATAAATAAATCCTGGTGTCCCT
GTTGATACCGGGAAGCCCTGGGCCAACTTTTGGCGAAAATGAGACGTTGATCGGCACGTAAGAGGTTCCAACTTTCA
CCATAATGAAATAAGATCACTACCGGGCGTATTTTTTGAGTTATCGAGATTTTCAGGAGCTAAGGAAGCTAAAATGG
AGAAAAAAATCACTGGATATACCACCGTTGATATATCCCAATGGCATCGTAAAGAACATTTTGAGGCATTTCAGTCA
GTTGCTCAATGTACCTATAACCAGACCGTTCAGCTGGATATTACGGCCTTTTTAAAGACCGTAAAGAAAAATAAGCA
CAAGTTTTATCCGGCCTTTATTCACATTCTTGCCCGCCTGATGAATGCTCATCCGGAATTCCGTATGGCAATGAAAG
ACGGTGAGCTGGTGATATGGGATAGTGTTCACCCTTGTTACACCGTTTTCCATGAGCAAACTGAAACGTTTTCATCG
CTCTGGAGTGAATACCACGACGATTTCCGGCAGTTTCTACACATATATTCGCAAGATGTGGCGTGTTACGGTGAAAA
CCTGGCCTATTTCCCTAAAGGGTTTATTGAGAATATGTTTTTCGTCTCAGCCAATCCCTGGGTGAGTTTCACCAGTT
TTGATTTAAACGTGGCCAATATGGACAACTTCTTCGCCCCCGTTTTCACCATGGGCAAATATTATACGCAAGGCGAC
AAGGTGCTGATGCCGCTGGCGATTCAGGTTCATCATGCCGTTTGTGATGGCTTCCATGTCGGCAGAATGCTTAATGA
ATTACAACAGTACTGCGATGAGTGGCAGGGCGGGGCGTAATTTTTTTAAGGCAGTTATTGGTGCCCTTAAACGCCTG
GTTGCTACGCCTGAATAAGTGATAATAAGCGGATGAATGGCAGAAATTCGAAAGCAAATTCGACCCGGTCGTCGGTT
CAGGGCAGGGTCGTTAAATAGCCGCTTATGTCTATTGCTGGTTTACCGGTTTATTGACTACCGGAAGCAGTGTGACC
GTGTGCTTCTCAAATGCCTGAGGCCAGGTACCGAGCTCGAATTCGTAATCATGTCATAGCTGTTTCCTGGCATAACC
TGATTCGTGGTATGTGGGTAACAAGTGTAATCTGTGTCACAATTCAGTCAGTTGACAGTTGCCTGTCAGACTGAGCA
TTTGTTAAAAAAATTTCGCATGGTGAATCCCCCTGTGTGGAGGGGCGACTGGTGAAAAATCCTTGCTTGTGATTCAT
TATCGACACGGGTTCGGTGGTACCAGGCCGAACTCAC…

B.

Supplementary Figure S6. Verification of *E. coli* O104:H4 *stx2::cat* by whole genome sequencing. **A.** Coverage of the BAM files generated by mapping sequencing reads using the *E. coli* O104:H4 genome GCF_000299455.1 as a reference genome and visualized in Integrative Genomics Viewer. No reads could be mapped to *stx2*. **B.** Sequence of the *stx2:: cat* region from the *de novo* assembly. The sequence of the insert carrying the resistance cassette (bold) flanked by 200 nt of the Stx2 phage region is shown. The *cat* ORF is marked in yellow and t-RNA O3K_RS16215 in green.

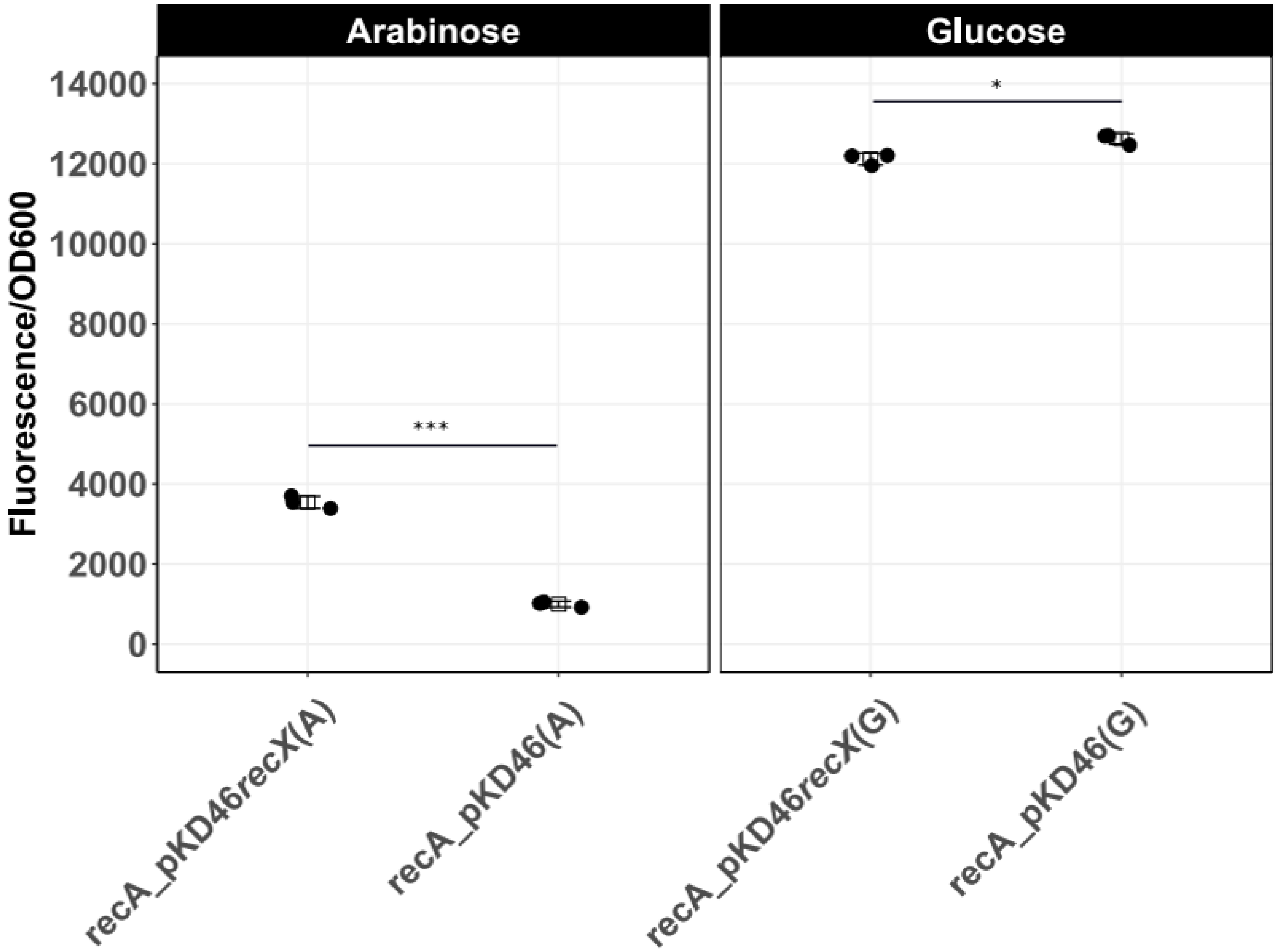

Supplementary Figure S7. Overall *E. coli* K-12 MG1655 SENSOR SOS response to sparfloxacin challenge. Shown are values and standard deviations of three biological replicates. Statistically significant t test comparisons are indicated by * ($p < 0.05$), *** ($p < 0.001$) and "ns" if not statistically significant.[1]

Supplementary Tables

Supplementary Table S1. Bacterial strains used in this study.

Strain	Construction / Reference
E. coli K-12 MG1655	[1]
EHEC O157:H7 EDL933 *Δstx1/stx2*	[2]
E. coli DH5*α*	[3]
E. coli K-12 MG1655 P*recA* SENSOR	this study

Supplementary Table S2. Plasmids used in this study.

Plasmid	Construction /Reference
pKD46	[4]
pKD46*recX*	this study
pLP1	this study
pLP2	this study

pWKSP*frr*	this study
pWKSP*frr-cfp-aph(3′)-Ia*	this study
pMB47	[5]
pMB54	[6]
pUC18	[7]
pWKS30	[8]

Supplementary Table S3. Primers used in this study.

Primer	Sequence (5′ - 3′)
MBP5	GGATGAATGGCAGAAATTCG
MBPD80	AACTGCAGCGCCGAATAAATACCTG
MBPD81	GGGGTACCTGGCCTCAGGCATTTGA
MBP223	TGTGACACAGATTACACTTGTTACCCACATACCACGAATCAGGTTAT GCCAGGAAACAGCTATGACCATG
MBP226	CTTTAGCTCAGTGGTGAGAG
MBP227	ACGAGTATCTCATGCAATTG
MBP260	CGAAGTGATCTTCCGTCACA
MBP272	TTCCCGTGATGGATAAATAAG
MBP273	GTTACGAATCCTTGAAAACTT
MBP274	TGTGTGTGACTGTCTGGTCTGACTGAGACAAGTTTTCAAGGATTCGTA ACATGGTGTCTATCACTAAAGATC
MBP275	TGAAGTTTTAAATCAATCTAAAGTATATATGAGTAAACTTGGTCTGAC AGAGGAAACAGCTATGACCATG
MBP276	CACAGGTTGCTCCGGGCTATGAAATAGAAAAATGAATCCGTTGAAGC CTGTTCCCGTGATGGATAAATAAG
MBP277	AACTTTTTGTCTTTTTACCTTCCCGTTTCGCTCAAGTTAGTATAAAAAA GAGGAAACAGCTATGACCATG
MBP278	GGTATTGATAATCCTGATATG
MBP279	GGCGCAATGCCATCTGGTAT
MBP280	GACGGGAAACTGAAAATGTG
MBP301	AGCTACAGGCGGTCAGCGTCACGCCAAAAGCCAATGCCAGCGCCAG ACGGACGACGTTGTAAAACGACGG
MBP302	CAACGGATTCATTTTTCTATTTCATAGCCCGGAGCAACCTGTGAACAC ATAGGAAACAGCTATGACCATGATTAC
MBP303	CGGCTCGTATGTTGTGTGGAATTGTGAGCGGATAACAATTTCACACA GGAACGACGTTGTAAAACGACGG
MBP304	GGCTTGCCCCGTGGTTTTCTGGCGTGCTGCGTAGTATCAGCGGCAATT ACAGGAAACAGCTATGACCATGATTAC
MBP305	TATGGCTCGCCATCAGGATC
MBP307	CGAATGGCGCAAAACCTTTCGCGGTATGGCATGATAGCGCCCGGAA GAGAACGACGTTGTAAAACGACGG
MBP308	TGGGGTGCCTAATGAGTGAGCTAACTCACATTAATTGCGTTGCGCTCA CTAGGAAACAGCTATGACCATGATTAC
MBP309	ATCTGCCAGTTTGAGGGGAC
MBP313	GAGTGGGACAAAATTGAAATC
MBP364	TCTGCGCCGGGTCTGGTGCTGATTACTTCAGCCAAAAGGAACACCTG TATACGACGTTGTAAAACGACGG

MC_204	TAAATGCTTCAATAATATTGAAAAAGGAAGAGTATGAGTATTCAACA TGCAGGAAACAGCTATGACCATG
MC_205	AACTTTTTGTCTTTTTACCTTCCCGTTTCGCTCAAGTTAGTATAAAAAA GTACTGTATGAGCATACAGTA

Supplementary step-by-step protocols

Preparation of recombination proficient cells

1. Store bacteria containing either pKD46 or pKD46*recX* at -80 °C. For the experiments, spread on a LB plate containing ampicillin [100 µg/ml final concentration(f.c.)] and incubated overnight t at 30 °C.
2. The next day, inoculate a single colony in 2 ml DYT medium containing ampicillin [100 µg/ml f.c.] and incubate at 180 rpm / 30 °C overnight.
3. Dilute 1:200 in 100 ml DYT medium containing ampicillin [100 µg/ml f.c.] and grow the cells at 180 rpm / 30 °C.
4. Measure $OD_{595\ nm}$ every hour.
5. When the $OD_{595\ nm}$ reaches 0.4, induce the expression of the enzymes by adding 3 ml 10 % L(+)-arabinose and incubate the culture at 180 rpm / 37 °C for another 1 h.
6. Transfer the culture on ice for 20 min.
7. Separate the cultures in ice-cold 50 ml Falcon tubes and harvest the culture at 1000 g / 4 °C for 15 min.
8. Decant supernatant, resuspend the pellet in 10 ml ice-cold H_2O and add ice-cold H_2O to 50 ml and collect the cells by centrifugation at 1000 g / 4 °C for 15 min.
9. Decant supernatant, resuspend the pellet in 10 ml ice-cold H_2O, unify the content of 2 Falcon tubes (continue with 1x 50 ml resuspended cells in 1 Falcon tube), add ice-cold H_2O to 50 ml and collect the cells by centrifugation at 1000 g / 4 °C for 15 min.
10. Decant supernatant, resuspend the pellet in 10 ml ice-cold H_2O, add ice-cold H_2O to 50 ml and collect the cells by centrifugation at 1000 g / 4 °C for 15 min; resuspend in an appropriate volume of ice-cold H_2O.
11. Measure OD_{595} of a 1:100 dilution; dilute with ice-cold H_2O in a way that a 1:100 dilution of the resuspendend cells has an $OD_{595\ nm}$ = 0.5.

12. Transfer 80 µl to micro cuvette to do a test for arcing; if arcing occurs, wash once more with ice-cold H_2O or 10.
13. Make 70 µl aliquots and electroporate the recombination substrate (here: Bio-Rad MicroPulser, setting "bacteria" in 0.1 cm Gene Pulser/Micro Pulser electroporation cuvettes)
14. Wash the cells out of the cuvette with 1 ml DYT (room temperature) and incubate for 1 h at 37 °C / shaking.
15. Harvest the cells by centrifugation at 1000 g / 5 min. Resuspend the pellet in 100 µl DYT and spread on an agar plate containing the appropriate antibiotic (here chloramphenicol 12.5 µg/ml f.c.)

Colony PCR

1. Pick colony from the plates and resuspend in 50 µl ddH_2O water. As a negative control, pick one corresponding wild type colony (containing either pKD46 or pKD46*recX*).
2. Incubate the suspension at 95 °C on a heating block and shake at 1400 rpm for 10 min.
3. Spin down debris at 11000 g for 5 min and place on ice. 5 µl of the supernatant is afterwards used as template in the PCR.

The standard recipe and program for our colony PCRs are:

**Reaction mixture**	**[µl]**
template	5
forward primer [10 µM]	1
reverse primer [10 µM]	1
2x GoTaq G2 Green Mastermix (Promega)	10
ddH_2O	3
Total volume	20

**PCR program:**		
95 °C	3 min	
95 °C	30 sec	30x
55 °C	30 sec	30x
72 °C	45 sec	30x
72 °C	5 min	
12 °C	hold	

References

1. Blattner, F.R.; Plunkett, G.; Bloch, C.A.; Perna, N.T.; Burland, V.; Riley, M.; Collado-Vides, J.; Glasner, J.D.; Rode, C.K.; Mayhew, G.F.; et al. The Complete Genome Sequence of Escherichia Coli K-12. *Science* **1997**, *277*, 1453–1462, doi:10.1126/science.277.5331.1453.

2. Gobert, A.P.; Vareille, M.; Glasser, A.-L.; Hindré, T.; Sablet, T. de; Martin, C. Shiga Toxin Produced by Enterohemorrhagic Escherichia Coli Inhibits PI3K/NF-KB Signaling Pathway in Globotriaosylceramide-3-Negative Human Intestinal Epithelial Cells. *The Journal of Immunology* **2007**, *178,* 8168–8174, doi:10.4049/jimmunol.178.12.8168.
3. Grant, S.G.; Jessee, J.; Bloom, F.R.; Hanahan, D. Differential Plasmid Rescue from Transgenic Mouse DNAs into Escherichia Coli Methylation-Restriction Mutants. *Proc Natl Acad Sci U S A* **1990**, *87*, 4645–4649, doi:10.1073/pnas.87.12.4645.
4. Datsenko, K.A.; Wanner, B.L. One-Step Inactivation of Chromosomal Genes in Escherichia Coli K-12 Using PCR Products. *Proc.Natl.Acad.Sci.U.S.A.* **2000**, *97*, 6640–6645, doi:10.1073/pnas.120163297.
5. Berger, M.; Aijaz, I.; Berger, P.; Dobrindt, U.; Koudelka, G. Transcriptional and Translational Inhibitors Block SOS Response and Shiga Toxin Expression in Enterohemorrhagic Escherichia Coli. *Sci Rep* **2019**, *9*, 18777, doi:10.1038/s41598-019-55332-2.
6. Berger, M.; Gerganova, V.; Berger, P.; Rapiteanu, R.; Lisicovas, V.; Dobrindt, U. Genes on a Wire: The Nucleoid-Associated Protein HU Insulates Transcription Units in Escherichia Coli. *Sci.Rep.* **2016**, *6*, 31512, doi:10.1038/srep31512.
7. Norrander, J.; Kempe, T.; Messing, J. Construction of Improved M13 Vectors Using Oligodeoxynucleotide-Directed Mutagenesis. *Gene* **1983**, *26*, 101–106, doi:10.1016/0378-1119(83)90040-9.
8. Rong Fu Wang; Kushner, S.R. Construction of Versatile Low-Copy-Number Vectors for Cloning, Sequencing and Gene Expression in Escherichia Coli. *Gene* **1991**, *100*, 195–199, doi:10.1016/0378-1119(91)90366-J.
